# Sphingosine‐1‐Phosphate Attenuates LPS‐Induced Inflammatory Cardiac Injury in Association With RASGRP1–S100A9–NLRP3 Signaling

**DOI:** 10.1002/ddr.70391

**Published:** 2026-09-22

**Authors:** Chaofu Yue, Qiaolin Li, Chunyan Li, Taoxian Yang, Xian Huang, Feng Yue, Qiuyu Long, Rong Li, Rong Lei, Qingsong Ma, Caimei Hu, Qian Yang, Yongjun Yan, Yuan Liu, QinYong Yan, Mei Yang

**Affiliations:** ^1^ Department of Intensive Care Unit Affiliated Qujing Hospital of Kunming Medical University, Qujing Central Hospital of Yunnan Province Qujing Yun Nan China; ^2^ Department of Endocrinology and Metabolism Affiliated Qujing Hospital of Kunming Medical University, Qujing Central Hospital of Yunnan Province Qujing Yun Nan China; ^3^ Nursing Department Affiliated Qujing Hospital of Kunming Medical University, Qujing Central Hospital of Yunnan Province Qujing Yun Nan China

**Keywords:** cardioprotection, inflammatory cardiac injury, lipopolysaccharide, NLRP3 inflammasome, sphingosine‐1‐phosphate (S1P)

## Abstract

Lipopolysaccharide (LPS) induces endotoxemia‐associated inflammatory cardiac injury rather than classical viral or autoimmune myocarditis. Sphingosine‐1‐phosphate (S1P) regulates cardiovascular and immune responses, predominantly through S1P receptors, but whether it also modulates macrophage‐associated inflammatory signaling during LPS‐induced cardiac injury remains incompletely understood. This study investigated the role of the RASGRP1–S100A9–NLRP3 signaling axis in the cardioprotective effects of S1P. Transcriptomic and single‐cell RNA‐sequencing analyses were used to identify inflammation‐associated candidate genes and their cellular distribution. Male Sprague‐Dawley rats received a single intraperitoneal injection of LPS (8 mg/kg) followed by S1P treatment for 6 weeks. Histopathology, echocardiography, serum cardiac‐injury markers, oxidative‐stress indices, inflammatory cytokines, Western blotting, and cellular metabolic‐flux assays were evaluated. RAW264.7 macrophages were used for mechanistic cellular studies. RASGRP1 or NLRP3 overexpression and S100A9‐R101Q mutation were used to assess functional contributions. Molecular docking and molecular‐dynamics simulations were complemented by SPR, MST, CETSA, DARTS, and Co‐IP assays to evaluate S1P–RASGRP1 target engagement and pathway‐associated protein interactions. Bioinformatic analyses identified RASGRP1 as a macrophage‐enriched, inflammation‐associated hub gene. In LPS‐treated rats, S1P reduced myocardial inflammatory injury and collagen deposition, improved ejection fraction and fractional shortening, and decreased cardiac‐injury, oxidative‐stress, and inflammatory markers. In RAW264.7 macrophages, S1P attenuated inflammatory and oxidative‐stress responses, restored oxidative phosphorylation and glycolytic capacity, and reduced the abundance of RASGRP1, S100A9, NLRP3, and ASC. SPR and MST supported a concentration‐dependent biophysical interaction between S1P and RASGRP1; CETSA and DARTS provided complementary target‐stability evidence. The molecular‐dynamics trajectory showed substantial rearrangement of S1P from the starting pose before reaching a plateau and therefore supports conformational sampling rather than preservation of the original docked pose. Co‐IP supported protein associations within the proposed inflammatory network. RASGRP1 or NLRP3 overexpression attenuated several S1P‐associated protective effects. The S100A9‐R101Q mutation also weakened several S1P‐associated protective responses. SPR indicated a lower apparent dissociation constant for R101Q than for WT S100A9 after model‐specific fitting, which is consistent with stronger or more persistent NLRP3 binding rather than loss of the S100A9–NLRP3 interaction. S1P mitigates LPS‐induced inflammatory cardiac injury in association with altered macrophage RASGRP1–S100A9–NLRP3 signaling. Biophysical and target‐stability assays support direct engagement of RASGRP1 by S1P. However, these findings do not establish RASGRP1 as the sole mediator of S1P activity, exclude contributions from canonical S1P receptor signaling, or demonstrate a strictly linear RASGRP1–S100A9–NLRP3 signaling cascade. Because the LPS model primarily represents endotoxemia‐associated inflammatory cardiac injury, extrapolation of these findings to classical viral or autoimmune myocarditis should be made with caution.

**Abbreviations**
CK‐MB: creatine kinase‐MB
cTnT: cardiac troponin T
DAMPs: danger‐associated molecular patterns
DEGs: differentially expressed genes
ECAR: extracellular acidification rate
EF: ejection fraction
FS: fractional shortening
LDH: lactate dehydrogenase
LPS: lipopolysaccharide
NLRP3: NOD‐like receptor family pyrin domain‐containing 3
OCR: oxygen consumption rate
RASGRP1: RAS guanyl‐releasing protein 1
ROC: receiver operating characteristic
S1P: sphingosine‐1‐phosphate
scRNA‐seq: single‐cell RNA sequencing

## 1 Introduction

Myocarditis is an inflammatory disorder of the myocardium and a major cause of sudden cardiac death and dilated cardiomyopathy, particularly in young adults (Weintraub et al. 2017). Although advances in diagnostic imaging have improved detection, inflammatory cardiac injury still accounts for a substantial proportion of unexplained acute cardiac dysfunction (Ammirati et al. 2020). Current therapeutic strategies remain largely supportive or immunosuppressive and lack targeted interventions against the underlying molecular mechanisms driving myocardial inflammation and injury (Soulaidopoulos et al. 2025).

Inflammatory myocardial injury reflects a complex interplay between injured cardiomyocytes and dysregulated immune responses, with macrophages serving as important mediators of innate inflammatory signaling (Sun et al. 2024; Wang and Han 2020). Both innate and adaptive immune cells infiltrate the myocardium, releasing pro‐inflammatory cytokines, reactive oxygen species, and danger‐associated molecular patterns (DAMPs) that amplify tissue injury (She et al. 2025; Silvis et al. 2020; Yang et al. 2023). Among these pathways, the nucleotide‐binding oligomerization domain‐like receptor family pyrin domain‐containing 3 (NLRP3) inflammasome has emerged as a central mediator of myocardial inflammation (Higashikuni et al. 2023; Zhang et al. 2021). Activation of NLRP3 leads to caspase‐1‐dependent maturation of interleukin‐1β and interleukin‐18, thereby promoting inflammatory cell recruitment, cardiomyocyte pyroptosis, and adverse cardiac remodeling (Liu et al. 2024; Narendran et al. 2020; Pan et al. 2024). Excessive NLRP3 activation has been implicated in multiple cardiac disorders, and pharmacological inhibition of inflammasome components has shown cardioprotective effects in preclinical models (Suceveanu et al. 2020).

Recent studies have identified RAS guanyl‐releasing protein 1 (RASGRP1) as a key regulator of macrophage pro‐inflammatory responses and intracellular inflammatory signaling, playing a central role in the excessive inflammation and oxidative stress associated with sepsis (Li et al. 2024). In parallel, accumulating evidence has highlighted‐ S100A9, a calcium‐binding alarmin predominantly released by activated myeloid cells, ‐ as a key amplifier of sterile inflammation (Uriepero‐Palma et al. 2025). Although both ‐RASGRP1 and S100A9 are implicated in myeloid inflammatory signaling, it remains unclear whether and how ‐RASGRP1 influences S100A9‐mediated responses. S100A9, often forming a heterodimer with S100A8, activates pattern recognition receptors such as Toll‐like receptor 4 (TLR4) and the receptor for advanced glycation end products (RAGE), triggering NF‐κB signaling and priming NLRP3 inflammasome activation (Chen et al. 2023a). Elevated S100A9 expression correlates with disease severity in inflammatory cardiac injury and other cardiovascular conditions, whereas experimental inhibition of S100A9 attenuates cardiac inflammation and remodeling (Chen et al. 2024; Marinković et al. 2020).

Sphingosine‐1‐phosphate (S1P) is a bioactive sphingolipid that regulates immune cell trafficking, vascular homeostasis, and inflammatory signaling through five G protein‐coupled receptors (S1PR1–S1PR5) (Obinata and Hla 2019; Wang et al. 2023). Dysregulated S1P signaling has been linked to cardiovascular diseases, including ischemic injury and heart failure, and growing evidence suggests that S1P intersects with inflammatory pathways involved in myocardial inflammation and fibrosis, positioning it as a potential modulator of inflammatory cardiac injury progression (Duan et al. 2024; Vestri et al. 2017). Although S1P is generally considered cardioprotective through mechanisms such as enhancing endothelial barrier function and promoting cardiomyocyte survival via S1PR1 signaling, emerging data suggest a dual role in inflammatory contexts (Chen et al. 2022; Wang et al. 2022). In macrophages and other innate immune cells, S1P/S1PR2 signaling can promote NLRP3 inflammasome priming and activation under conditions of inflammatory stimulation, linking S1P receptor signaling with key pro‐inflammatory pathways (Tian et al. 2025). This receptor‐specific divergence underscores the complexity of S1P biology in immune modulation and suggests that the net effect of S1P in inflammatory cardiac injury may depend on the balance of receptor subtype expression and local inflammatory cues.

Despite growing evidence for the immunomodulatory role of S1P, whether S1P‐associated cardioprotection is accompanied by modulation of macrophage‐associated RASGRP1–S100A9–NLRP3 signaling remains unknown. We hypothesized that S1P would attenuate LPS‐induced inflammatory cardiac injury while suppressing this macrophage‐associated inflammatory network. We also evaluated whether S1P directly engages RASGRP1, while recognizing that canonical S1PR‐dependent signaling remains a biologically plausible parallel or interacting mechanism. The LPS model was therefore used as a controlled system for intense innate immune activation and myocardial injury and was not intended to reproduce the full etiological or adaptive‐immune features of classical viral or autoimmune myocarditis.

## 2 Materials and Methods

### 2.1 Acquisition and Preprocessing of Public Cardiac Transcriptomic Data

Publicly available cardiac transcriptomic data were obtained from the Gene Expression Omnibus (GEO) database. GSE35182, generated using the Affymetrix Mouse Gene 1.0 ST Array platform, contains cardiac gene‐expression profiles from coxsackievirus B3 (CVB3)‐infected mice and corresponding uninfected controls and was used as a discovery dataset for myocarditis‐associated inflammatory signatures. Raw CEL files were imported into R (version 4.2.0) and processed using the robust multi‐array average (RMA) procedure, including background correction, quantile normalization, and log_2_‐scale expression summarization. Probe sets were mapped to mouse gene symbols using the corresponding platform annotation, probes without valid gene annotations were removed, and multiple probes mapping to the same gene were summarized to a single gene‐level expression value. After preprocessing and annotation, 17,962 gene‐level variables were retained for differential‐expression analysis.

Differential expression between CVB3‐infected and control samples was assessed using linear models with empirical Bayes moderation implemented in the limma package. *p* values were adjusted for multiple comparisons using the Benjamini–Hochberg method. Genes meeting the criteria of adjusted *p* < 0.05 and |log_2_ fold change| > 1 were considered significantly differentially expressed genes (DEGs). Gene Ontology (GO) and Kyoto Encyclopedia of Genes and Genomes (KEGG) enrichment analyses of the significant DEGs were performed using the clusterProfiler package together with the mouse‐specific annotation database org.Mm.eg.db. Enrichment results were visualized using bar plots, dot plots, and Circos plots.

### 2.2 Identification of Macrophage‐Associated Differentially Expressed Genes

Given the critical involvement of macrophages in myocarditis pathogenesis, macrophage‐associated genes (MAGs) were retrieved from the Molecular Signatures Database (MSigDB, version 7.5.1). The C5 Gene Ontology (GO) collection was queried using the keyword “macrophage,” yielding a curated set of macrophage‐associated genes. The intersection between myocarditis DEGs (from GSE35182) and MAGs was identified using Venn diagram analysis. These overlapping genes were defined as macrophage‐enriched differentially expressed genes (MA‐DEGs). MA‐DEGs were subsequently used for downstream protein–protein interaction (PPI) network construction and pathway enrichment analyses to identify key macrophage‐associated hub genes involved in the inflammatory response of myocarditis.

### 2.3 Protein–Protein Interaction Network Construction and Functional Enrichment Analysis

A PPI network of MA‐DEGs was constructed using the STRING database (version 11.5) with a minimum interaction confidence score of 0.7. The resulting network was imported into Cytoscape (version 3.9.1) for visualization and topological analysis. Key hub genes were identified based on network parameters, including degree, betweenness, and closeness centrality. Among these, RASGRP1 was identified as a core macrophage‐enriched hub gene in myocarditis. Functional characterization of MA‐DEGs was performed using GO and KEGG enrichment analyses, revealing significant enrichment in pro‐inflammatory signaling pathways, as well as GO terms related to interferon‐γ response and leukocyte adhesion, consistent with a pervasive pro‐inflammatory transcriptional program in myocarditis.

### 2.4 Single‐Cell Rna Sequencing Analysis and RASGRP1 Expression Profiling

Single‐cell RNA‐sequencing (scRNA‐seq) data from cardiac tissues were obtained and analyzed through the Home for Researchers platform (https://www.home-for-researchers.com/#/). Available dataset‐source and sample metadata on the platform were reviewed before analysis. Quality control, normalization, and scaling were performed using Seurat (v4.1.0) in R. Highly variable genes were identified, followed by principal component analysis (PCA) for dimensionality reduction. Cells were clustered using a graph‐based algorithm, and t‐distributed stochastic neighbor embedding (t‐SNE) was applied for two‐dimensional visualization of cellular heterogeneity. Cell clusters were annotated based on canonical marker genes, identifying major cardiac cell populations, including monocytes/macrophages, cardiomyocytes, endothelial cells, and T cells. The expression of RASGRP1 was mapped across cell populations using feature and violin plots, revealing predominant localization in macrophages. Correlation analyses linked RASGRP1 expression to inflammation scores and pro‐inflammatory genes, while pathway enrichment analysis of RASGRP1‐high macrophage clusters supported an inflammation‐associated transcriptional phenotype.

### 2.5 Rats and Animal Procedures

Male Sprague‐Dawley (SD) rats (8 weeks old, 250–300 g) were obtained from GemPharmatech Co. Ltd. (Nanjing, China). All animal experiments were approved by Kunming Medical University and conducted in accordance with the National Institutes of Health Guide for the Care and Use of Laboratory Animals. Rats were housed under specific pathogen‐free conditions at 22 ± 2°C with a 12‐h light/dark cycle and were allowed free access to standard chow and water.

To investigate inflammation‐driven myocardial injury in vivo, we employed an LPS‐induced inflammatory cardiac injury model. This model was selected to reproduce robust innate immune activation, oxidative stress, myocardial injury, and cardiac dysfunction under controlled experimental conditions. LPS (Sigma‐Aldrich, USA) was dissolved in sterile saline and administered intraperitoneally at 8 mg/kg as a single challenge; control rats received an equal volume of sterile saline. The model is intended to represent endotoxemia‐associated inflammatory cardiac injury and does not reproduce viral infection, cardiac antigen‐specific adaptive immunity, or the complete pathophysiology of classical viral or autoimmune myocarditis.

Rats were randomly assigned to four groups (*n* = 6 per group): (1) Control (saline); (2) LPS model; (3) LPS + S1P (Sphingosine‐1‐phosphate, 25 mg/kg, Sigma‐Aldrich, USA); (4) LPS + S1P (50 mg/kg). S1P was dissolved in DMSO, diluted with 0.9% sterile saline, and administered intraperitoneally once daily for 6 consecutive weeks after LPS injection. Cardiac function was evaluated by echocardiography, and serum biochemical analyses were performed to assess myocardial injury (CK‐MB, LDH, cTnT) and inflammatory responses (IL‐6, TNF‐α, IL‐10). Heart tissues and blood samples were collected for subsequent histological, molecular, and biochemical analyses. To investigate the functional contribution of S100A9 Arg101, AAV‐S100A9‐WT and AAV‐S100A9‐R101Q constructs were packaged into AAV9 vectors (GeneChem, China) and administered by tail vein injection (1 × 10^12^ viral genomes per rat) 2 weeks before LPS challenge. The 6‐week treatment and follow‐up period was chosen to assess outcomes beyond the initial inflammatory insult, including collagen deposition, structural remodeling, and cardiac recovery; it does not imply persistent LPS exposure or acute endotoxemia throughout the 6 weeks.

### 2.6 AAV Vector Construction and Administration

To investigate the mechanistic roles of RASGRP1 and NLRP3 in inflammatory cardiac injury, adeno‐associated virus serotype 9 (AAV9) vectors were employed for in vivo gene delivery. Recombinant AAV9 vectors carrying the following constructs were obtained from a commercial supplier:

(1) AAV‐NC (empty vector control);

(2) AAV‐RASGRP1‐WT (wild‐type RASGRP1 overexpression);

(3) AAV‐NLRP3 (NLRP3 overexpression).

Each rat received 1 × 10^12^ viral genome particles (vg) via tail‐vein injection 14 days before LPS challenge to ensure stable transgene expression. Successful transgene overexpression was confirmed by Western blot analysis of cardiac tissues. No overt toxicity or adverse effects were observed following AAV administration.

### 2.7 Cell Transfection

To investigate the functional roles of RASGRP1 and NLRP3, RAW264.7 macrophages were transduced with recombinant AAV vectors carrying empty control (AAV‐NC), RASGRP1 overexpression (AAV‐RASGRP1), or NLRP3 overexpression (AAV‐NLRP3). Cells were seeded at appropriate density and incubated with AAV particles for 24 h, followed by replacement with fresh medium. After 48 h to allow stable gene expression, cells were stimulated with LPS (1 μg/mL) and treated with S1P (40 μM) as indicated. Overexpression efficiency was verified by Western blot analysis before subsequent oxidative stress, inflammatory, and mitochondrial metabolic assays.

### 2.8 Virtual Screening of S1P Targeting RASGRP1

To identify potential small‐molecule compounds targeting RASGRP1, a hierarchical virtual screening strategy was conducted using the HY‐L021P natural product library (6017 compounds). The three‐dimensional structure of RASGRP1 was obtained from the Protein Data Bank and prepared using the Protein Preparation Wizard module in Schrödinger, including hydrogen addition, charge assignment, and energy minimization. Molecular docking was carried out using the Glide module, sequentially applying high‐throughput virtual screening (HTVS), standard precision (SP), and extra precision (XP) docking protocols. Compounds were ranked according to docking scores, and the top candidates were retained for further analysis. Among the final hits, S1P exhibited the highest predicted binding affinity to RASGRP1 and was therefore selected for subsequent experimental validation.

### 2.9 Target Prediction and Pharmacological Network Analysis of S1P in Myocarditis

Potential molecular targets of S1P relevant to myocarditis were identified using an integrated multi‐database computational approach. The three‐dimensional structure of S1P was retrieved from PubChem and submitted to SwissTargetPrediction, PharmMapper, and DrugBank to predict putative S1P‐associated targets. After removal of duplicate entries, unique target genes were retained. Associated genes were collected from GeneCards and OMIM databases. The intersection between predicted S1P targets and myocarditis‐associated genes was identified using Venn diagram analysis. These overlapping genes were considered putative therapeutic targets of S1P in myocarditis.

A PPI network of the intersecting genes was constructed using the STRING database (confidence score ≥ 0.7) and visualized with Cytoscape. Network topological parameters, including degree, betweenness, and closeness centrality, were calculated to identify hub genes. To explore the biological functions and signaling pathways associated with S1P in myocarditis, GO and KEGG enrichment analyses were performed using the clusterProfiler R package. Enrichment results, including biological processes, molecular functions, cellular components, and signaling pathways, were visualized using bubble plots and bar plots generated in R, highlighting pro‐inflammatory and immune regulatory pathways consistent with the results described in Section 3.

### 2.10 Molecular Docking and Binding Mode Analysis

Molecular docking analyses were performed to characterize the interactions between S1P and key proteins in the RASGRP1–NLRP3–S100A9 axis. The three‐dimensional structures of RASGRP1 and S100A9 were obtained from the Protein Data Bank (PDB) and prepared by removing water molecules, adding hydrogen atoms, and optimizing side‐chain conformations. Docking simulations were conducted using AutoDock Vina 1.2.0, and top‐ranked binding poses were selected based on docking scores and binding conformations. Docking poses were analyzed using PyMOL and Discovery Studio Visualizer to identify key interactions, including hydrogen bonds, hydrophobic contacts, and spatial complementarity within the protein binding pockets. These docking results guided subsequent site‐directed mutagenesis and functional validation experiments.

### 2.11 Histological Staining and Pathological Assessment of Cardiac Tissue

Rat hearts were fixed in 4% paraformaldehyde (Servicebio, China) for 24 h, embedded in paraffin, and serially sectioned at a thickness of 5 μm. Hematoxylin and eosin (H&E) staining (Solarbio, China) was performed to evaluate myocardial architecture, cardiomyocyte degeneration, interstitial edema, inflammatory cell infiltration, and necrotic injury. Masson's trichrome staining (Solarbio, China) and Sirius Red staining (Sigma, USA) were used to assess myocardial fibrosis and collagen deposition, respectively. Myocardial histological injury was semi‐quantitatively evaluated using a 0–4 ordinal scoring system, adapted from previously reported scoring criteria for experimental LPS‐associated myocardial injury:

0, normal myocardial architecture without detectable injury;

1, mild injury characterized by interstitial edema and focal cardiomyocyte degeneration or necrosis;

2, moderate injury characterized by diffuse cardiomyocyte swelling/degeneration with multifocal myocardial necrosis;

3, severe injury characterized by extensive myocardial necrosis, contraction‐band changes, and prominent inflammatory cell infiltration;

4, very severe injury characterized by widespread or confluent myocardial necrosis accompanied by marked leukocyte infiltration, contraction‐band changes, and/or interstitial hemorrhage. The histological injury score is an ordinal, dimensionless value, with higher scores indicating more severe myocardial damage. Histological sections were evaluated across multiple non‐overlapping myocardial fields, and the mean score obtained from the evaluated fields was used as the representative injury score for each animal. Quantitative image analysis was performed using ImageJ software. For Masson's trichrome staining, myocardial fibrosis was expressed as the percentage of collagen‐positive area relative to the total myocardial tissue area. Similarly, Sirius Red‐positive collagen deposition was quantified as the percentage of positively stained area relative to the total myocardial area. Scale bars and magnifications are provided in the corresponding figure panels and legends.

### 2.12 Assessment of Inflammatory Activity and Immune‐Cell Infiltration

Inflammatory activity and immune‐cell infiltration were estimated from the normalized transcriptomic expression matrix using single‐sample gene set enrichment analysis (ssGSEA) implemented in the GSVA R package. Predefined inflammation‐associated gene signatures and immune cell‐specific gene sets were used to quantify the relative enrichment of inflammatory processes and individual immune‐cell populations in each sample. For each sample, genes were ranked according to their expression levels, and ssGSEA calculated an enrichment score reflecting the coordinated expression of genes within each predefined signature. The inflammation score was therefore defined as the ssGSEA enrichment score of the inflammation‐associated gene set. Similarly, macrophage and monocyte infiltration was estimated using macrophage‐ and monocyte‐specific gene signatures, respectively.

These scores are dimensionless relative enrichment measures and should not be interpreted as absolute cell counts or percentages of immune cells. Higher inflammation scores indicate greater relative enrichment of the inflammatory transcriptional program, whereas higher macrophage or monocyte scores indicate greater relative enrichment of the corresponding immune‐cell‐associated gene signature. The resulting enrichment scores were compared between CVB3‐infected myocarditis and uninfected control samples and were used for subsequent correlation analyses with RASGRP1 expression. Because animals were followed for 6 weeks after the initial LPS challenge, Masson's trichrome and Sirius Red staining were additionally performed to assess subsequent myocardial remodeling, fibrosis, and collagen deposition at the study endpoint.

### 2.13 Echocardiographic Assessment

Transthoracic echocardiography was performed using a Vevo 2100 high‐resolution ultrasound system (VisualSonics, Canada). Rats were lightly anesthetized with 1%–2% isoflurane. Two‐dimensional and M‐mode recordings were obtained from parasternal long‐axis and short‐axis views. Left ventricular systolic function was assessed by measuring ejection fraction (EF) and fractional shortening (FS).

### 2.14 Immunohistochemistry

IL‐6 expression in cardiac tissues was examined by immunohistochemical staining. Paraffin‐embedded sections were deparaffinized, rehydrated, and subjected to heat‐mediated antigen retrieval in citrate buffer. Endogenous peroxidase activity was quenched with 3% hydrogen peroxide. Sections were incubated with anti‐IL‐6 antibody (Proteintech, USA; 21865‐1‐AP, 1:200) overnight at 4°C, followed by incubation with HRP‐conjugated secondary antibody (CST, USA; #7074, 1:500) for 1 h at room temperature. Staining was developed using a DAB chromogenic kit (Boster, China) and counterstained with hematoxylin.

### 2.15 Enzyme‐Linked Immunosorbent Assay (ELISA)

Serum and cell culture supernatants were collected to measure inflammatory cytokines (IL‐6, #H007‐1‐1, TNF‐α, # H052‐1‐1, IL‐10, # H009‐1‐1) using ELISA kits (Proteintech, USA) according to manufacturer's instructions (Nanjing Jiancheng Bioengineering Institute).

### 2.16 Creatine Kinase MB Isoenzyme (CK‐MB) and Cardiac Troponin (cTnT) Assay

Serum concentrations of CK‐MB and cTnT were determined using the Creatine Kinase MB Isoenzyme Assay Kit (H197–1‐2) and the Cardiac Troponin Assay Kit (H149‐4‐1) from Nanjing Jiancheng Bioengineering Institute (Nanjing, China), following the manufacturer's instructions. Absorbance was measured at 450 nm using a microplate spectrophotometer.

### 2.17 Measurement of Oxidative Stress Markers

Cardiac tissues and cultured cells were homogenized in ice‐cold phosphate‐buffered saline (PBS) and centrifuged to obtain the supernatants for biochemical analysis. The activities of superoxide dismutase (SOD; A001‐3‐2), glutathione peroxidase (GSH‐Px; A005‐1‐2), and catalase (CAT; A007‐1‐1), as well as the content of malondialdehyde (MDA; A003‐1‐2), were determined using commercially available assay kits according to the manufacturer's instructions (Nanjing Jiancheng Bioengineering Institute, China). Absorbance was measured using a spectrophotometer (Thermo Fisher Scientific, USA).

### 2.18 Western Blot Analysis

Total protein was extracted from frozen heart tissues and cultured cells using RIPA lysis buffer (Beyotime, China) supplemented with protease and phosphatase inhibitor cocktails (MCE, China). Protein concentrations were determined using a BCA protein assay kit (Beyotime, China). Equal amounts of protein were separated by SDS–PAGE and subsequently transferred onto polyvinylidene fluoride (PVDF) membranes (Millipore, USA). After blocking with 5% non‐fat milk in Tris‐buffered saline containing 0.1% Tween‐20 (TBST) for 1 h at room temperature, membranes were incubated overnight at 4°C with primary antibodies against RASGRP1 (Proteintech, USA; 26997‐1‐AP, 1:1000), S100A9 (Proteintech, USA; 26992‐1‐AP, 1:1000), NLRP3 (Proteintech, USA; 30109‐1‐AP, 1:1000), and ASC (Immunoway, USA; YM8352, 1:1000). After washing with TBST 3 times, membranes were incubated with HRP‐conjugated secondary antibodies (CST, USA) for 1 h at room temperature. Protein bands were visualized using an enhanced chemiluminescence (ECL) detection system (Bio‐Rad, USA) and quantified using ImageJ software. β‐Actin (TransGen Biotech, China; HC201, 1:3000) or GAPDH (Proteintech, USA; 60004‐1‐Ig, 1:2000) served as internal loading controls.

### 2.19 Cell Culture

RAW264.7 murine macrophages (ATCC, USA) were cultured in DMEM (Gibco, USA) supplemented with 10% fetal bovine serum (FBS, Gibco, USA) and 1% penicillin–streptomycin (Gibco, USA) at 37°C in a humidified incubator with 5% CO_2_. Cells were seeded in six‐well plates and allowed to reach 70%–80% confluence before treatments. To establish an in vitro LPS‐induced macrophage inflammatory model, RAW264.7 cells were first primed with LPS (1 μg/mL; Escherichia coli O111:B4, Sigma‐Aldrich, USA) for 24 h. For anti‐inflammatory treatment, S1P (40 μM; Sigma‐Aldrich, USA) was administered 24 h prior to LPS stimulation. Control cells received an equivalent volume of vehicle (DMSO, <0.1%). After treatments, cells and culture supernatants were collected for downstream molecular, biochemical, and immunological analyses.

### 2.20 Cell Viability and Lactate Dehydrogenase Release

Cell viability was evaluated using the Cell Counting Kit‐8 (CCK‐8) assay (MCE, China) following the manufacturer's instructions. Cells were seeded in 96‐well plates at a density of 1 × 10^4^ cells per well and incubated overnight at 37°C in a humidified atmosphere with 5% CO_2_. After treatment, 10 μL of CCK‐8 reagent was added to each well and incubated for an additional 1 h. Absorbance at 450 nm was measured using a microplate reader (Thermo, USA).

LDH release was measured as an indicator of cellular injury using a commercial kit (Beyotime, China). Briefly, 120 μL of culture supernatant was collected and transferred to a new 96‐well plate. LDH reaction mixture was added according to the manufacturer's instructions and incubated for 30 min. Absorbance was recorded at 490 nm using a microplate reader (Thermo, USA).

### 2.21 Mitochondrial Respiration and Glycolytic Function (OCR and ECAR)

Mitochondrial function in RAW264.7 macrophages was assessed using the Seahorse XF96 Extracellular Flux Analyzer (Agilent, USA). Cells were seeded at 2 × 10^4^ cells per well in Seahorse XF96 microplates and incubated overnight. Prior to measurement, the culture medium was replaced with Seahorse XF assay medium supplemented with 10 mM glucose, 1 mM pyruvate, and 2 mM l‐glutamine, and cells were incubated in a CO_2_‐free incubator at 37°C for 1 h. Oxygen consumption rate (OCR) and extracellular acidification rate (ECAR) were measured under basal conditions and after sequential injections of oligomycin (1 μM), FCCP (1 μM), and rotenone/antimycin A (0.5 μM). Mitochondrial parameters including basal respiration, maximal respiration, ATP production, and spare respiratory capacity were calculated. Glycolysis parameters, including glycolysis rate, glycolytic capacity, and glycolytic reserve, were assessed using the Seahorse Glycolysis Stress Test Kit (Agilent, USA), consistent with the metabolic analyses described in the results.

### 2.22 Bioinformatics and Network Pharmacology Analysis

To predict potential therapeutic targets of S1P in myocarditis, bioinformatics and network pharmacology analyses were performed. S1P‐associated targets were retrieved from the SwissTargetPrediction, PharmMapper, and DrugBank databases. Myocarditis‐associated genes were collected from the GeneCards and OMIM databases. Overlapping genes between predicted S1P targets and myocarditis‐associated genes were identified using Venn diagram analysis and considered as putative therapeutic targets of S1P. A PPI network of overlapping genes was constructed using the STRING database (confidence ≥ 0.7) and visualized with Cytoscape 3.9.1. Network topological parameters, including degree, betweenness, and closeness centrality, were calculated to identify hub genes. GO functional annotation and KEGG pathway enrichment analysis of hub genes were performed using the clusterProfiler R package, and results were visualized with bubble and bar plots, consistent with the transcriptional and pathway analyses described in the results.

### 2.23 Molecular Docking

The experimental structure of human RASGRP1 used for S1P docking was obtained from the Protein Data Bank (PDB ID: 4L9M). This structure is an unliganded, autoinhibited RASGRP1 structure and does not contain a biologically relevant co‐crystallized ligand. Crystallization additives and nonessential water molecules were removed, polar hydrogens and charges were assigned, missing side‐chain atoms were repaired where required, and the structural Zn^2+^ ions in the RASGRP1 C1 domain were retained. Because no native ligand is present in 4L9M, native‐ligand re‐docking and comparison with a RASGRP1 co‐crystal reference‐ligand score were not applicable. S100A9 and NLRP3 structures were used separately for hypothesis‐generating protein–protein interface analyses associated with the Arg101 mutation experiments.

The chemical identity and stereochemistry of S1P were reverified using PubChem CID 5283560. A three‐dimensional structure was generated from the verified isomeric representation, its stereochemistry was retained, physiologically relevant protonation was assigned, and the ligand was energy‐minimized before docking. The corrected S1P structure was used for the revised docking analysis and figure preparation.

Docking was performed using AutoDock Vina 1.2.0 with the search space centered on the predicted RASGRP1 binding pocket. Exhaustiveness was set to 32, 20 poses were generated, and an energy range of 4 kcal/mol was used. Candidate poses were evaluated according to Vina score, absence of major steric clashes, and interactions within the predicted pocket. Because RASGRP1 PDB 4L9M lacks a biologically relevant co‐crystallized ligand, docking validation relied on transparent acknowledgment of this structural limitation, molecular‐dynamics evaluation of pose stability, and experimental target‐engagement assays rather than an inappropriate native‐ligand re‐docking comparison. The selected RASGRP1–S1P complex was subjected to a 100‐ns molecular‐dynamics simulation using GROMACS. The solvated system was neutralized, adjusted to 0.15 M NaCl, energy‐minimized, and equilibrated under NVT and NPT conditions before the production run. Complex stability was evaluated using protein‐backbone RMSD, ligand RMSD, residue‐level RMSF, hydrogen‐bond occupancy, and interaction persistence. Binding free‐energy analysis was performed using an MM/PBSA approach on equilibrated trajectory frames. These computational analyses were interpreted as support for the stability of a predicted binding configuration rather than as independent proof of direct molecular binding.

### 2.24 Machine Learning‐Assisted Feature Selection and Candidate Gene Prioritization

To prioritize robust candidate genes associated with inflammatory myocardial disease, three complementary feature‐selection approaches—recursive feature elimination (RFE), random forest (RF), and least absolute shrinkage and selection operator (LASSO) logistic regression—were applied to the candidate‐gene expression matrix. RFE iteratively removed less informative features, RF analysis used the randomForest package in R to rank feature importance, and LASSO logistic regression was performed using glmnet with lambda. min as the selected penalty parameter. Genes selected by at least two of the three approaches were treated as consensus candidates for subsequent ROC analysis, network analysis, and experimental prioritization. These analyses are described as machine learning‐assisted feature selection and candidate‐gene prioritization rather than autonomous target identification.

### 2.25 Surface Plasmon Resonance (SPR)

Surface plasmon resonance (SPR) was used to characterize the direct biophysical interactions between S1P and RASGRP1 and between S100A9 and NLRP3. For S1P–RASGRP1 binding analysis, recombinant RASGRP1 was immobilized on the sensor surface, and S1P was injected over a series of concentrations. Sensorgrams were background‐corrected and analyzed to assess concentration‐dependent binding and estimate the apparent equilibrium dissociation constant (K_D). For analysis of the S100A9–NLRP3 interaction, recombinant S100A9‐WT or S100A9‐R101Q was examined for binding to NLRP3 over the indicated concentration range. Association and dissociation responses were fitted using appropriate kinetic or concentration–response models to obtain apparent binding parameters. All binding analyses were performed independently of the molecular docking predictions.

### 2.26 Microscale Thermophoresis (MST)

Microscale thermophoresis (MST) was performed as an orthogonal solution‐phase assay to further characterize the interaction between S1P and RASGRP1. RASGRP1 was incubated with serially diluted concentrations of S1P, and changes in thermophoretic behavior were monitored under identical measurement conditions. The normalized thermophoretic response was plotted against S1P concentration, and the resulting concentration–response curve was fitted to estimate the apparent dissociation constant (K_D). MST was used together with SPR to provide complementary biophysical characterization of S1P–RASGRP1 binding.

### 2.27 Cellular Thermal Shift Assay (CETSA)

The cellular thermal shift assay (CETSA) was performed to determine whether S1P altered the thermal stability of RASGRP1 in a protein‐containing experimental system. Samples were treated with S1P or vehicle control and subsequently exposed to a defined temperature gradient. After heating, aggregated proteins were removed by centrifugation, and the soluble protein fractions were collected. RASGRP1 abundance in the soluble fractions was determined by Western blotting, and the relative thermal stability of RASGRP1 was compared between S1P‐treated and control samples.

### 2.28 Drug Affinity Responsive Target Stability (DARTS)

Drug affinity responsive target stability (DARTS) analysis was performed to assess whether S1P altered the susceptibility of RASGRP1 to proteolytic degradation. Protein samples were pre‐incubated with S1P or vehicle control and subsequently subjected to limited proteolysis using Pronase E under controlled conditions. The digestion reaction was terminated at the indicated time point, and residual RASGRP1 protein was analyzed by Western blotting. Differences in RASGRP1 abundance after protease treatment were used to evaluate S1P‐associated changes in proteolytic stability.

### 2.29 Co‐Immunoprecipitation (Co‐IP)

Co‐immunoprecipitation (Co‐IP) was performed to examine protein associations among RASGRP1, S100A9, and NLRP3 in the macrophage experimental system. Cell lysates were prepared under non‐denaturing conditions and incubated with antibodies against the indicated target proteins, followed by capture of the immune complexes with protein A/G beads. Normal IgG was used as a negative immunoprecipitation control. After extensive washing, the precipitated protein complexes were eluted and analyzed by Western blotting for the corresponding interacting proteins. Reciprocal Co‐IP assays were performed to evaluate the associations between RASGRP1 and S100A9 and between S100A9 and NLRP3. In addition, RASGRP1‐containing complexes were immunoprecipitated from cells subjected to the indicated treatments and subsequently analyzed for co‐associated S100A9 and NLRP3. Input lysates were analyzed in parallel to verify protein expression.

### 2.30 Statistical Analysis

All experimental data were expressed as means ± SD. Statistical analyses were performed using GraphPad Prism 9 (GraphPad Software, USA). Comparisons among multiple groups were conducted using one‐way analysis of variance (ANOVA) with Tukey's post hoc test. Comparisons between two groups were performed using an unpaired two‐tailed Student's *t*‐test. Differences were considered statistically significant at *p* < 0.05, consistent with the significance thresholds applied in the results section.

## 3 Results

### 3.1 RASGRP1 Is a Macrophage‐Enriched Hub Gene in Myocarditis‐Associated Inflammatory Transcriptomic Data

**Figure 1:**
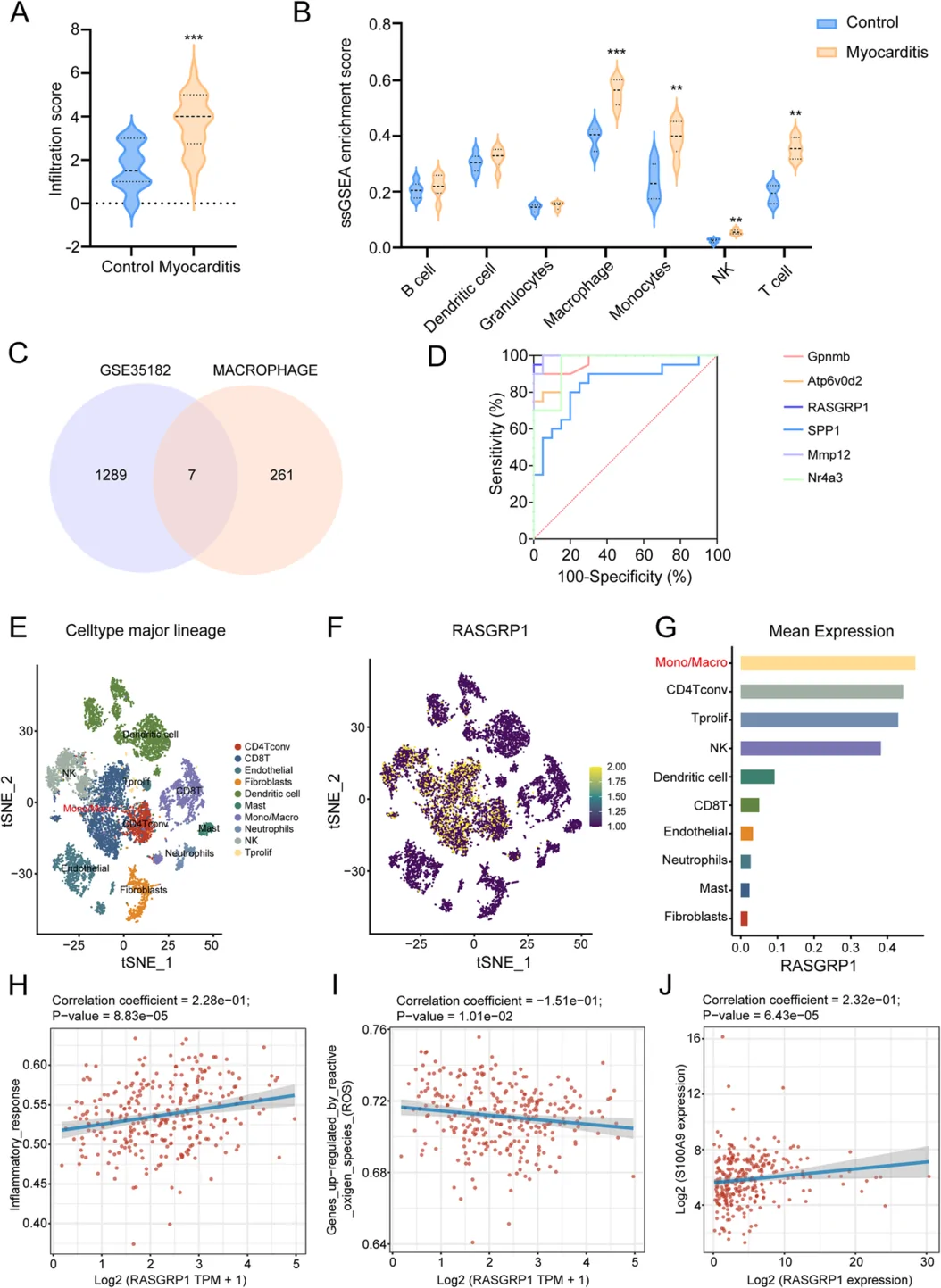
RASGRP1 is a macrophage‐enriched hub gene in inflammation‐associated cardiac transcriptomic data. (A) Violin plot comparing ssGSEA‐derived inflammation scores between CVB3 myocarditis and uninfected control samples. The values are dimensionless relative enrichment scores. (B) Violin plots showing immune‐cell‐associated ssGSEA enrichment scores, with relative enrichment of macrophage and monocyte signatures in CVB3 myocarditis. These scores are not percentages or absolute cell counts. (C) Venn diagram illustrating the overlap between DEGs from GSE35182 and a macrophage signature gene set (7 shared genes). (D) ROC curves evaluating the diagnostic performance of candidate genes (Gpnmb, Pdgfb, RASGRP1, Spp1, Mmp12, Nlrp3). (E) t‐SNE plot of major cardiac cell lineages, annotated by cell type. (F–G) *t*‐SNE and violin plots showing RASGRP1 expression, confirming its specific enrichment in macrophages. (H–J) Correlation analyses linking RASGRP1 expression to inflammation scores, pro‐inflammatory gene set enrichment, and target gene expression. Statistical analyses were performed using the Mann–Whitney *U* test, Student's *t*‐test, and Pearson correlation. Significance levels are indicated as *p < 0.05, **p < 0.01, and ***p < 0.001.

To characterize the inflammatory landscape and identify key molecular drivers, we first analyzed the GSE35182 mouse CVB3 myocarditis dataset. Inflammation scores were significantly higher in CVB3‐infected samples than in uninfected controls (Figure 1A). Immune‐cell signature analysis further showed relative enrichment of macrophage‐ and monocyte‐associated transcriptional programs in the infected samples (Figure 1B).

To identify macrophage‐associated hub genes, significant DEGs from GSE35182 were intersected with a macrophage signature gene set, yielding seven overlapping candidates (Figure 1C). ROC analysis showed that RASGRP1 had discriminatory performance within this discovery dataset (Figure 1D). Single‐cell RNA‐sequencing analysis was then used to assess cellular distribution. t‐SNE visualization of major cardiac cell lineages (Figure 1E), together with feature and violin plots (Figure 1F,G), showed that RASGRP1 expression was predominantly enriched in macrophages. Correlation analyses further associated RASGRP1 expression with inflammation scores and multiple pro‐inflammatory transcriptional features (Figure 1H–J). These data support a macrophage‐associated context for the subsequent cellular experiments and do not establish a macrophage‐associated RASGRP1 mechanism.

**Figure 2:**
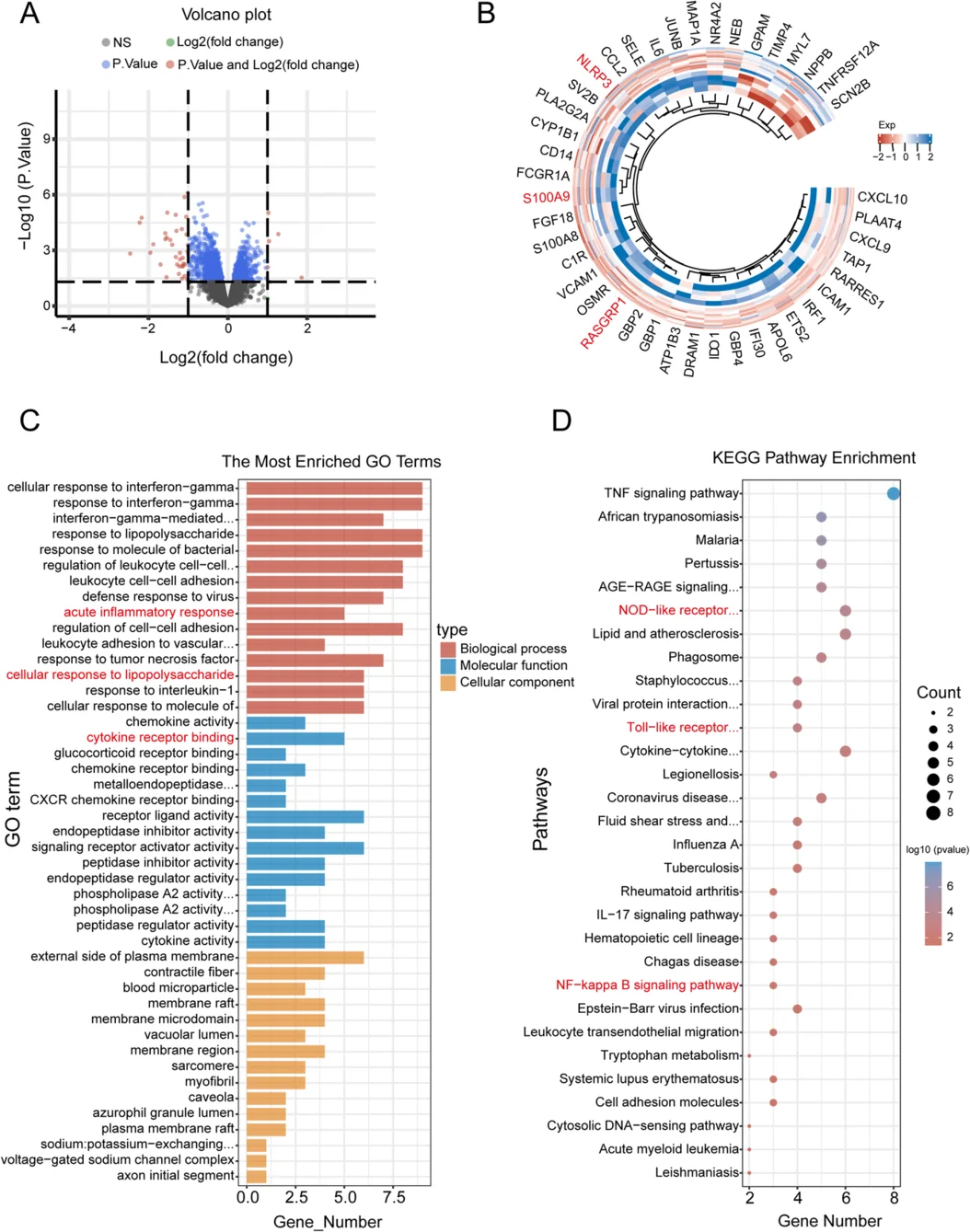
Transcriptomic profiling of the GSE35182 myocarditis dataset. (A) Volcano plot of 17,962 gene‐level variables included in the differential‐expression analysis. The value 17,962 denotes the total variables tested, not the number of significant DEGs; 42 genes met the predefined significance criteria. (B) Circos plots illustrated the expression patterns of key inflammatory mediators. (C, D) Functional enrichment analysis revealed the top Gene Ontology (GO) terms across biological process, cellular component, and molecular function categories, while KEGG pathway analysis highlighted enriched signaling pathways, with bubble size representing gene count and color indicating significance.

At the transcriptomic level, 17,962 gene‐level variables were included in the differential‐expression analysis; this number represents the total variables tested, not the number of significant DEGs. After the predefined adjusted‐*p*‐value and fold‐change thresholds were applied, 42 genes were identified as significantly differentially expressed between CVB3‐infected and control samples (Figure 2A). A Circos plot illustrated expression patterns of selected inflammatory mediators (Figure 2B). Functional enrichment analysis showed that these DEGs were significantly enriched in Gene Ontology (GO) terms related to interferon‐γ response and leukocyte adhesion (Figure 2C), as well as in KEGG pathways including TNF signaling, NOD‐like receptor signaling, and NF‐κB signaling (Figure 2D). Collectively, these results indicate that RASGRP1 represents a macrophage‐enriched hub gene closely associated with a pervasive pro‐inflammatory transcriptional program.

### 3.2 Identification of RASGRP1‐Targeting Compounds and Delineation of Its Inflammatory Network in Inflammatory Cardiac Injury

**Figure 3:**
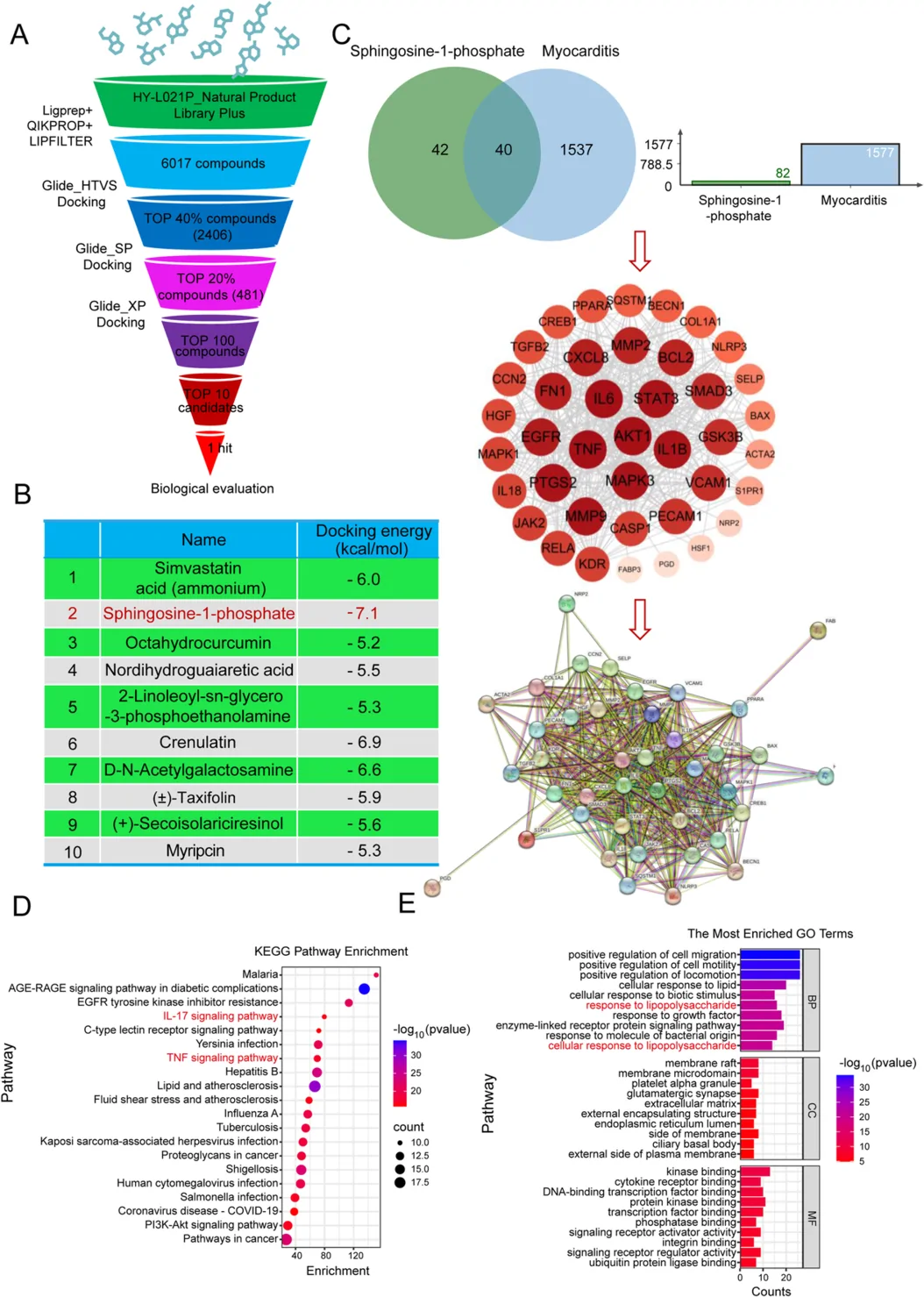
Virtual screening prioritizes S1P as a predicted RASGRP1‐binding candidate and defines an inflammation‐associated RASGRP1 network. (A) A multi‐stage virtual screening pipeline was applied to the HY‐L021P natural product library, sequentially filtered through Glide HTV, SP, and XP docking, yielding 10 candidate compounds. (B) The top 10 compounds were ranked by docking energy (kcal/mol), with Sphingosine‐1‐phosphate showing the highest predicted binding affinity. (C) A Venn diagram displays the overlap between RASGRP1‐correlated genes and myocarditis‐associated DEGs, identifying 40 shared genes. (C) Protein‐protein interaction (PPI) analysis of these 40 genes shows network connectivity, with node size corresponding to degree. A co‐expression network centered on RASGRP1 visualizes significant gene‐gene correlations. (D) The global PPI network of the RASGRP1 interactome is annotated by KEGG pathway enrichment. KEGG pathway enrichment is further presented as a bubble plot, where bubble size represents gene count and color indicates significance (−log_10_ *p* value). (E) The most significantly enriched Gene Ontology (GO) terms are displayed in a bar plot, categorized by Biological Process (blue), Cellular Component (red), and Molecular Function (purple), with color intensity reflecting −log_10_ *p* value.

To prioritize candidate compounds predicted to interact with RASGRP1, we performed a multi‐stage virtual screening workflow (Figure 3A). The HY‐L021P natural product library, comprising 6017 compounds, was subjected to sequential Glide HTVS, SP, and XP docking, resulting in 10 high‐affinity candidate molecules selected for further experimental validation. Among these candidates, sphingosine‐1‐phosphate (S1P) ranked highest based on docking scores (Figures 3B and S1). To further contextualize RASGRP1 within inflammation‐associated transcriptional networks, genes correlated with RASGRP1 expression were intersected with disease‐associated DEGs, identifying 40 shared genes (Figure 3C). Protein–protein interaction (PPI) network analysis revealed that these genes formed a tightly interconnected module, which was subsequently expanded into a global RASGRP1‐centered co‐expression network (Figure 3C). Functional enrichment analysis demonstrated that this network was significantly enriched in pro‐inflammatory signaling pathways, including TNF and NF‐κB signaling (Figure 3D), as well as GO terms associated with inflammatory and immune responses (Figure 3E).

**Figure 4:**
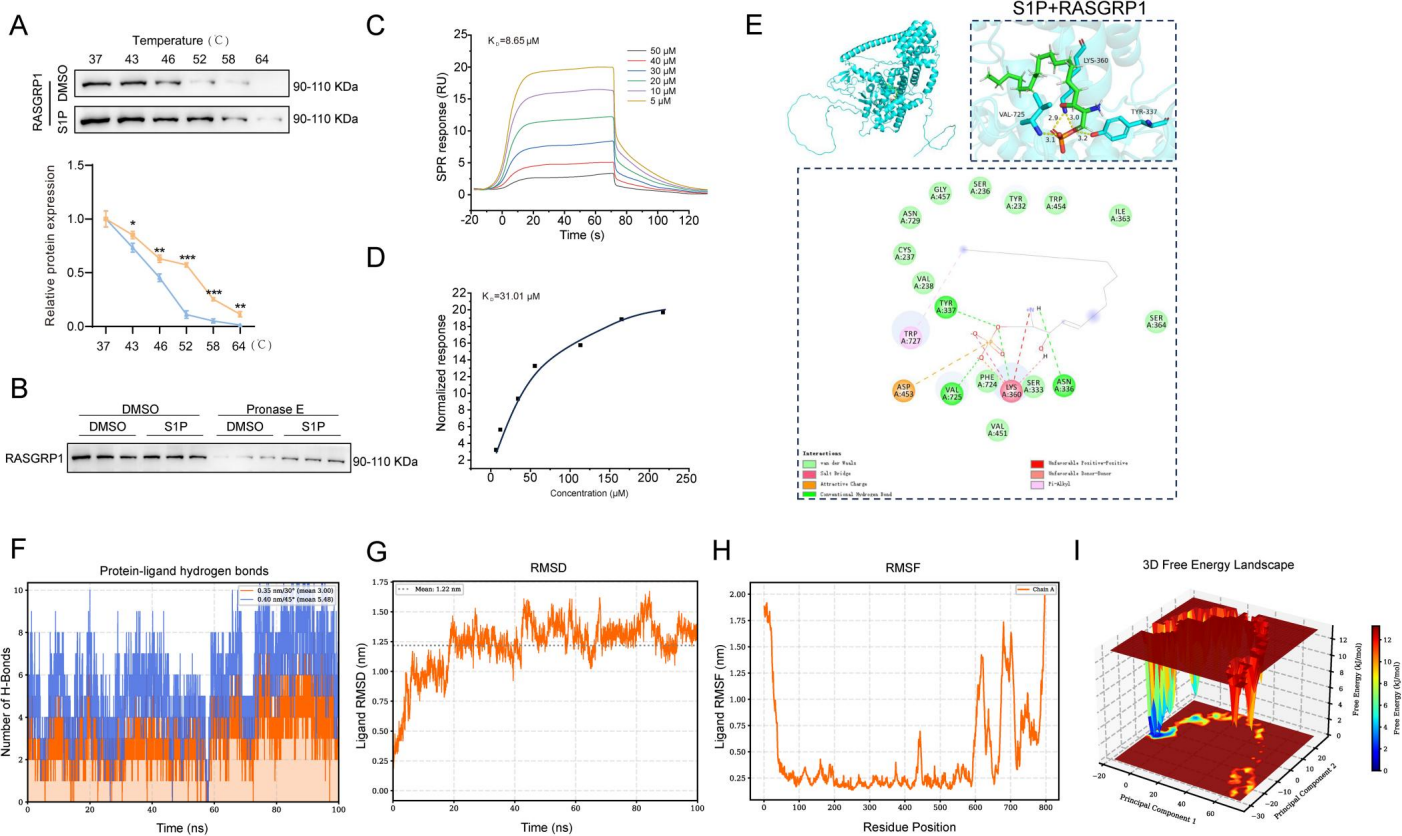
Experimental and computational validation of the interaction between S1P and RASGRP1. (A) CETSA showing the thermal stability of RASGRP1 in the presence of S1P or DMSO control at the indicated temperatures. (B) DARTS assay showing the resistance of RASGRP1 to Pronase E‐mediated proteolysis in the presence or absence of S1P. (C) SPR analysis of the interaction between S1P and RASGRP1, showing concentration‐dependent binding with an apparent equilibrium dissociation constant (K_D) of 8.65 μM. (D) MST analysis independently confirming the interaction between S1P and RASGRP1, with an apparent K_D of 31.01 μM. (E) Representative molecular docking model of S1P within the predicted binding pocket of RASGRP1, illustrating potential hydrogen‐bonding and noncovalent interactions with surrounding amino acid residues. (F) Number of hydrogen bonds formed between S1P and RASGRP1 during the 100‐ns molecular dynamics simulation under the indicated geometric criteria. (G) RMSD of S1P during the molecular dynamics simulation, reflecting the conformational stability of the ligand within the RASGRP1 binding environment. (H) RMSF analysis showing residue‐level structural fluctuations of the S1P–RASGRP1 complex during the simulation. (I) Free‐energy landscape of the S1P–RASGRP1 complex, illustrating the distribution of energetically favorable conformational states during molecular dynamics simulation.

To further validate the interaction between S1P and RASGRP1, complementary biochemical, biophysical, and computational approaches were employed. CETSA showed that S1P treatment increased the thermal stability of RASGRP1, as evidenced by greater retention of RASGRP1 protein at elevated temperatures compared with the DMSO control (Figure 4A). Consistently, DARTS analysis demonstrated that RASGRP1 was more resistant to Pronase E‐mediated proteolysis in the presence of S1P, further supporting ligand‐induced stabilization of RASGRP1 (Figure 4B). Direct binding was subsequently evaluated using two orthogonal biophysical assays. SPR revealed a clear concentration‐dependent binding response between S1P and RASGRP1, with an apparent equilibrium dissociation constant (K_D) of 8.65 μM (Figure 4C). MST independently confirmed the interaction and yielded an apparent K_D of 31.01 μM (Figure 4D), providing additional evidence for direct molecular recognition between S1P and RASGRP1.

Molecular docking further predicted that S1P could be accommodated within a binding pocket of RASGRP1 and stabilized by a network of hydrogen‐bonding and noncovalent interactions with surrounding residues (Figure 4E). Molecular dynamics simulations were subsequently performed to assess the dynamic stability of the predicted S1P–RASGRP1 complex. Multiple protein–ligand hydrogen bonds were maintained throughout the 100‐ns simulation, with mean hydrogen‐bond numbers of approximately 3.00 and 5.48 under the respective geometric criteria (Figure 4F). The ligand RMSD gradually reached a relatively stable regime during the simulation, with a mean value of approximately 1.22 nm, suggesting equilibration of the ligand within the protein environment after the initial conformational adjustment (Figure 4G). RMSF analysis showed relatively limited fluctuations across most regions, although increased flexibility was observed at several terminal or locally flexible regions (Figure 4H). Moreover, the free‐energy landscape displayed distinct low‐energy conformational basins, indicating the presence of energetically favorable conformational states of the S1P–RASGRP1 complex during the simulation (Figure 4I). Collectively, these biochemical, biophysical, and computational data provide convergent evidence supporting direct target engagement between S1P and RASGRP1 and the dynamic stability of the predicted S1P–RASGRP1 complex, while the precise residue‐level binding mechanism requires further experimental validation.

### 3.3 Sphingosine‐1‐phosphate (S1P) Alleviates LPS‐Induced Cardiac Injury and Cardiac Dysfunction in Rats

**Figure 5:**
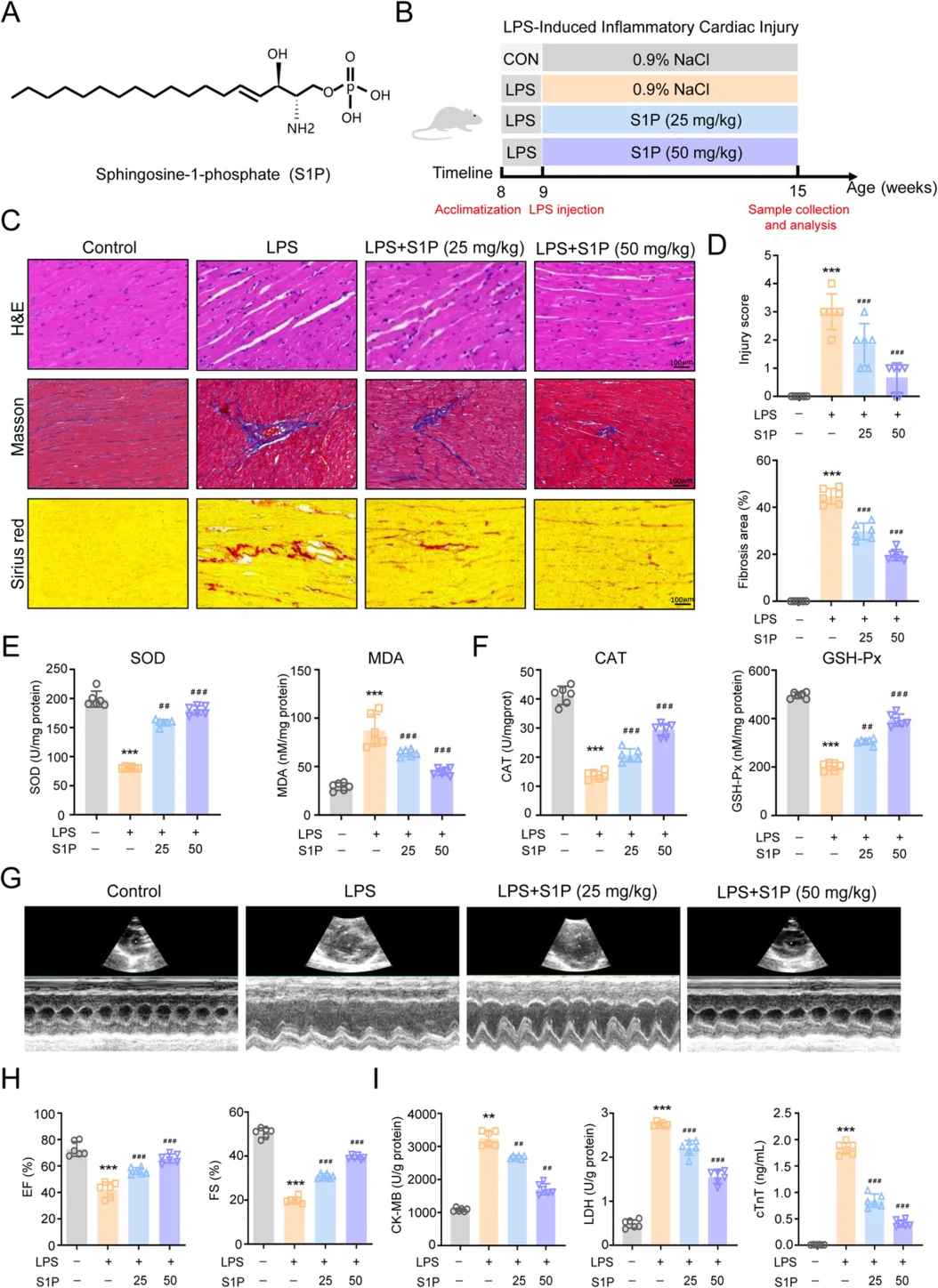
S1P alleviates LPS‐induced inflammatory cardiac injury and cardiac dysfunction in rats. (A) Chemical structure of Sphingosine‐1‐phosphate (S1P). (B) Experimental timeline and group design. A single intraperitoneal LPS challenge (8 mg/kg) initiated the inflammatory cardiac injury model, followed by daily S1P treatment at 25 or 50 mg/kg for six weeks; the prolonged follow‐up was used to assess post‐inflammatory remodeling and does not indicate persistent LPS exposure. (C) Representative H&E, Masson's trichrome, and Sirius Red‐stained myocardial sections obtained at the 6‐week endpoint. H&E staining was used to assess myocardial structural injury and inflammatory infiltration, whereas Masson's trichrome and Sirius Red staining were used to evaluate post‐inflammatory fibrosis and collagen deposition. Scale bar = 100 μm. (D) Quantitative injury and remodeling indices. (E, F) Myocardial oxidative stress markers, including superoxide dismutase (SOD), malondialdehyde (MDA), catalase (CAT), and glutathione peroxidase (GSH‐Px). (G) Representative M‐mode echocardiograms. (H) Quantification of left ventricular ejection fraction (EF) and fractional shortening (FS). (I) Serum levels of myocardial injury markers, including creatine kinase MB (CK‐MB), lactate dehydrogenase (LDH), and cardiac troponin T (cTnT). Data are presented as mean ± SD, with statistical significance indicated as *p < 0.05, **p < 0.01, ***p < 0.001 versus Control group; ^#^ *p* < 0.05, ^##^ *p* < 0.01, ^###^ *p* < 0.001 versus LPS group.

Based on the identification of S1P as a potential RASGRP1‐targeting compound, we next evaluated its therapeutic efficacy in an LPS‐induced inflammatory cardiac injury model (Figure 5A). Animals were randomly assigned to four groups: Control, LPS, and LPS co‐treated with S1P at doses of 25 or 50 mg/kg (Figure 5B).

Histopathological examination using hematoxylin and eosin (H&E), Masson's trichrome, and Sirius Red staining demonstrated that S1P administration dose‐dependently attenuated LPS‐induced myocardial inflammation, fibrosis, and collagen deposition (Figure 5C). Quantitative pathological scoring further confirmed significant reductions in both inflammatory infiltration and fibrotic remodeling following S1P treatment (Figure 5D). In parallel, S1P markedly alleviated LPS‐induced oxidative stress, as evidenced by restored activities of the antioxidant enzymes superoxide dismutase (SOD), catalase (CAT), and glutathione peroxidase (GSH‐Px), along with a concomitant reduction in malondialdehyde (MDA) levels (Figure 5E,F). Functional assessment by echocardiography revealed that S1P significantly improved LPS‐impaired cardiac performance, as indicated by increased ejection fraction (EF) and fractional shortening (FS) (Figure 5G,H). Consistent with these structural and functional improvements, S1P treatment dose‐dependently reduced serum levels of myocardial injury biomarkers, including creatine kinase‐MB (CK‐MB), lactate dehydrogenase (LDH), and cardiac troponin T (cTnT) (Figure 5I). Moreover, S1P effectively suppressed LPS‐induced elevations in pro‐inflammatory cytokines (IL‐6 and TNF‐α) while enhancing the anti‐inflammatory cytokine IL‐10 (Figure S2).

Collectively, these results demonstrate that S1P confers robust cardioprotective effects against LPS‐induced inflammatory cardiac injury by mitigating inflammatory injury, oxidative stress, and cardiac dysfunction, thereby supporting its therapeutic potential in inflammatory cardiac disease.

### 3.4 S1P Attenuates the RASGRP1–S100A9–NLRP3‐associated Inflammasome Pathway and Restores Mitochondrial Function in LPS‐Treated RAW264.7 Macrophages

**Figure 6:**
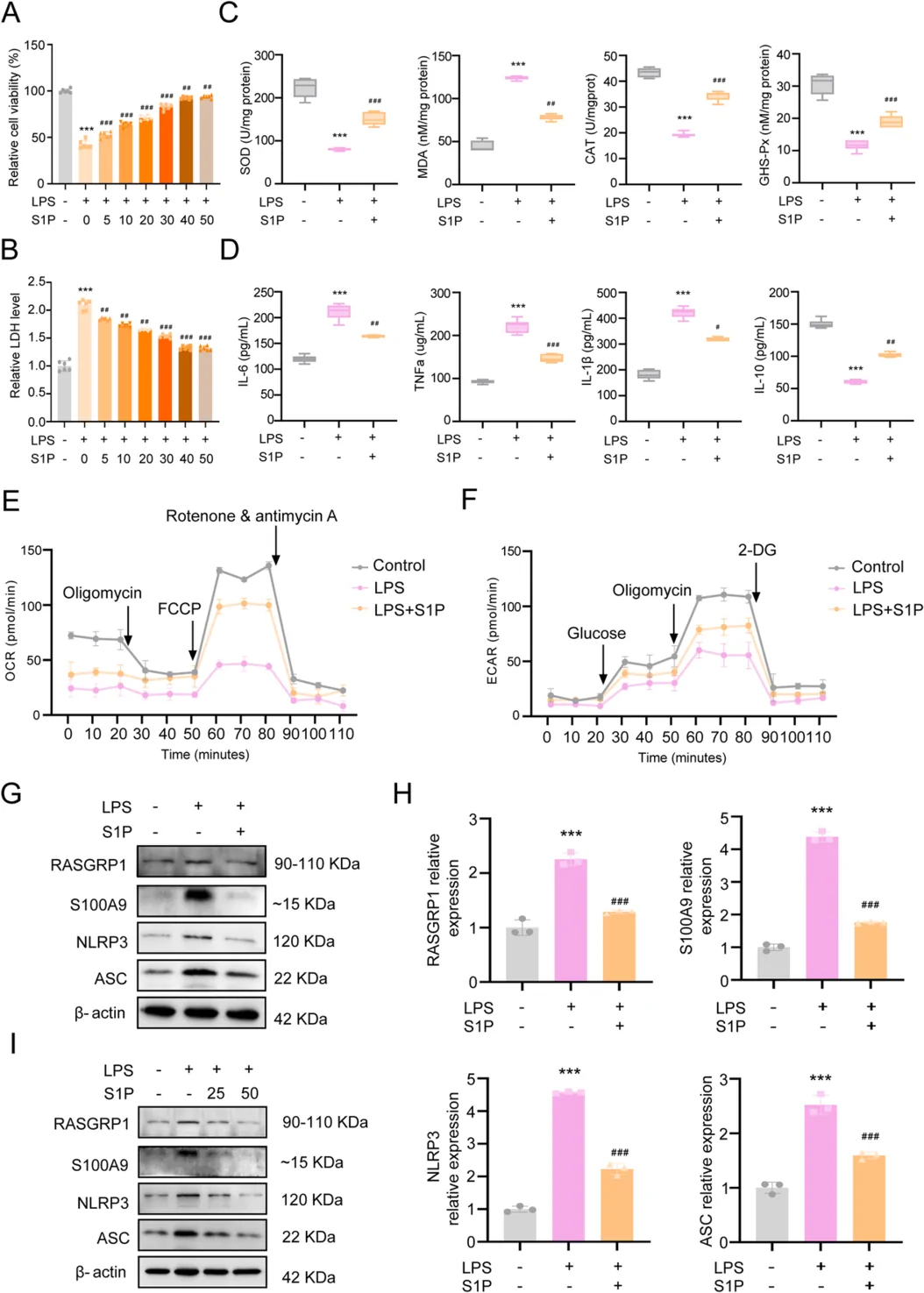
S1P attenuates macrophage inflammatory signaling and restores metabolic function in LPS‐treated RAW264.7 macrophages. (A) Cell viability assessed by CCK‑8 assay. (B) LDH release. (C) Antioxidant enzyme activities (SOD, CAT, GSH‑Px) and MDA levels. (D) Cytokine levels (IL‑6, TNF‑α, IL‑10, and IL‐1β） in culture supernatants. (E) Mitochondrial oxygen consumption rate (OCR) profile following sequential injection of oligomycin, FCCP, and rotenone/antimycin A. (F) Extracellular acidification rate (ECAR) profile after sequential injection of glucose, oligomycin, and 2‑DG. (G) Western blot analysis of RASGRP1, S100A9, NLRP3, ASC, and β‑actin under increasing S1P doses, with (H) quantification of protein levels and (I) representative blots for 25 and 50 μM S1P treatments. Data are expressed as mean ± SD, with statistical significance indicated as *p < 0.05, **p < 0.01, ***p < 0.001 versus Control group, and ^#^ *p* < 0.05, ^##^ *p* < 0.01, ^###^ *p* < 0.001 versus LPS‐treated group.

To examine macrophage‐associated mechanisms accompanying the in vivo cardioprotective phenotype, we assessed LPS‐induced inflammatory and metabolic dysfunction in RAW264.7 macrophages. LPS treatment dose‐dependently reduced macrophage viability and increased LDH release, whereas co‐treatment with S1P significantly reversed these detrimental effects (Figure 6A,B). Based on dose–response analyses, a concentration of 40 μM S1P was selected for subsequent experiments. In parallel, S1P markedly restored LPS‐impaired antioxidant capacity, as evidenced by increased activities of SOD, CAT, and GSH‐Px, along with a reduction in MDA levels (Figure 6C). Consistent with the in vivo findings, S1P significantly suppressed the LPS‐induced secretion of pro‐inflammatory cytokines IL‐6 and TNF‐α, while enhancing the release of the anti‐inflammatory cytokine IL‐10 (Figure 6D). Moreover, cellular levels of myocardial injury markers, including CK‐MB, LDH, and cTnT, were also markedly reduced following S1P treatment, further supporting the protective effects of S1P against LPS‐induced cellular inflammatory injury (Figure S3A).

To assess the impact of S1P on cellular energy metabolism, mitochondrial function was evaluated using Seahorse metabolic flux analysis. LPS exposure significantly impaired mitochondrial oxidative phosphorylation, as indicated by reductions in basal respiration, maximal respiration, and ATP production, and also suppressed glycolytic capacity, as reflected by decreased extracellular acidification rate (ECAR). Notably, all of these metabolic disturbances were substantially rescued by S1P treatment (Figures 6E,F and S3B,C). At the molecular level, S1P reduced the abundance of RASGRP1, S100A9, NLRP3, and ASC in LPS‐stimulated RAW264.7 macrophages (Figure 6G–I and Figure S4). These results demonstrate that S1P protects RAW264.7 macrophages from LPS‐induced injury by suppressing the RASGRP1–NLRP3 inflammasome axis, alleviating inflammatory and oxidative stress, and restoring mitochondrial metabolic function.

### 3.5 NLRP3 Overexpression Partially Attenuates S1P‐Associated Protective Effects

**Figure 7:**
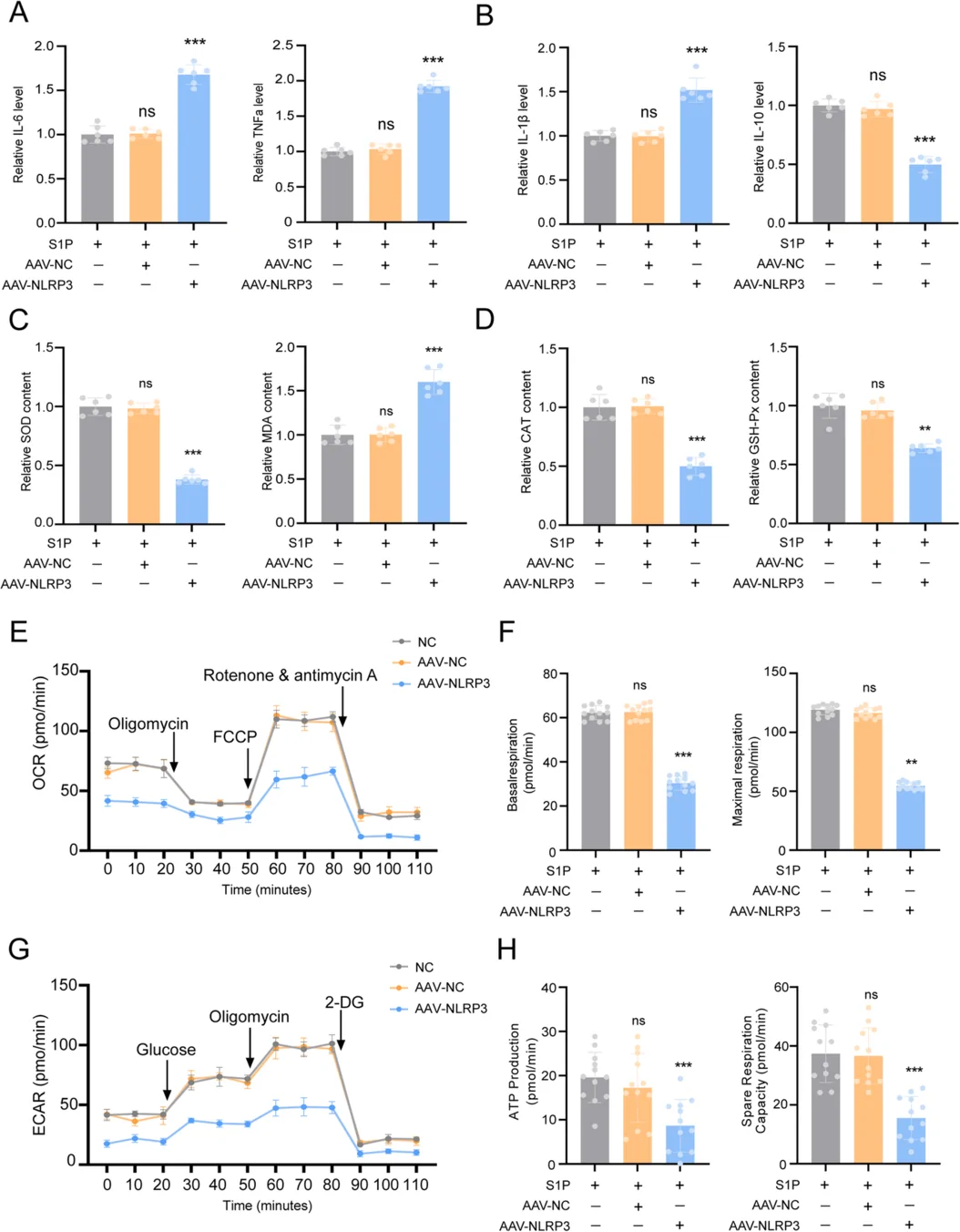
NLRP3 overexpression abrogates the cardioprotective effects of S1P. (A, B) Myocardial levels of IL‐6, TNF‐α, IL‐1β and IL‐10. (C, D) Antioxidant enzyme activities (SOD, CAT, GSH‐Px) and MDA levels. (E) Mitochondrial oxygen consumption rate (OCR) profile. (F) Quantification of basal and maximal respiration. (G, H) Extracellular acidification rate (ECAR) profile with quantification of ATP production and spare respiratory capacity. Data are presented as mean ± SD, with statistical significance indicated as **p < 0.01, ***p < 0.001 versus AAV‐NC + SIP group.

To determine whether the cardioprotective effects of S1P are mediated through the RASGRP1–NLRP3 axis, NLRP3 was overexpressed using adeno‐associated virus (AAV) in the LPS‐induced inflammatory cardiac injury model. Under these conditions, S1P treatment markedly suppressed LPS‐induced elevations in the pro‐inflammatory cytokines IL‐6 and TNF‐α while increasing the anti‐inflammatory cytokine IL‐10; however, these anti‐inflammatory effects were largely abolished upon NLRP3 overexpression (Figure 7A,B). Consistently, the S1P‐mediated restoration of antioxidant defenses—including increased activities of SOD, CAT, and GSH‐Px—as well as the reduction in lipid peroxidation, as indicated by decreased MDA levels, were significantly attenuated in the presence of NLRP3 overexpression (Figure 7C,D). Mitochondrial function analysis revealed that S1P rescued the LPS‐induced decline in oxidative phosphorylation and glycolytic capacity, improvements which were abrogated upon NLRP3 overexpression (Figures 7E–H and S5). These findings support a functional contribution of NLRP3 to the observed phenotype but do not by themselves establish NLRP3 as a strictly downstream component of a linear RASGRP1–S100A9–NLRP3 cascade.

### 3.6 RASGRP1 Overexpression Aggravates LPS‐Associated Cardiac Injury and Attenuates the Protective Phenotype of S1P

**Figure 8:**
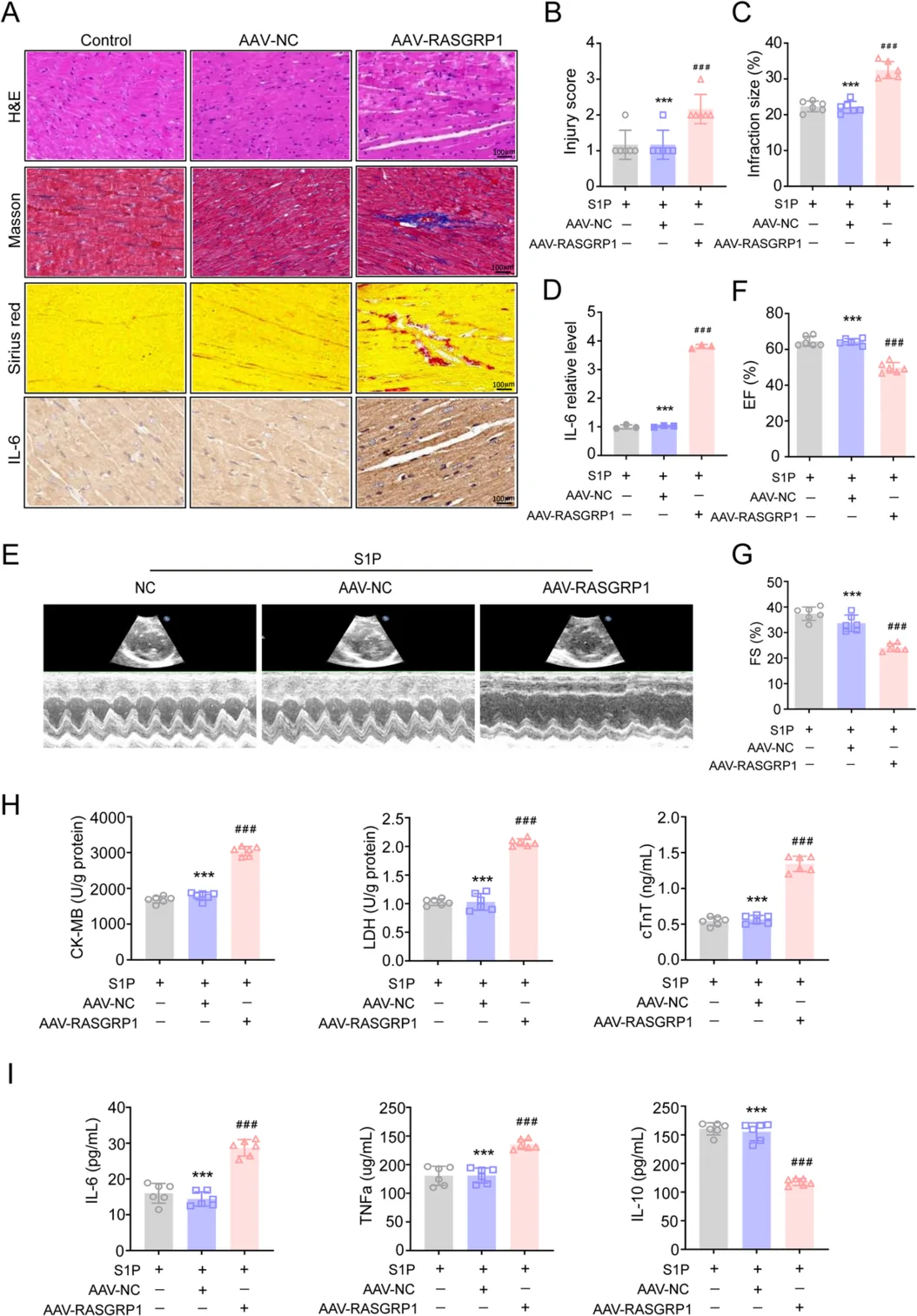
RASGRP1 overexpression exacerbates LPS‐induced inflammatory cardiac injury and attenuates several S1P‐associated improvements. (A) Representative myocardial histology (H&E, Masson's trichrome, Sirius Red) and IL‑6 immunohistochemistry. (B, C) Quantitative analysis of injury score and infarct size. (D) IL‐6 level. (E) Representative M‐mode echocardiograms. (F, G) Left ventricular ejection fraction (EF) and fractional shortening (FS). (H) Serum levels of CK‑MB, LDH, and cTnT. (I) Serum cytokine levels (IL‑6, TNF‑α, IL‑10). Data are presented as mean ± SD, with **p < 0.01, ***p < 0.001 versus NC + SIP group.

RASGRP1 overexpression markedly aggravated LPS‐induced myocardial pathological injury, as evidenced by enhanced inflammatory infiltration, increased fibrosis and collagen deposition, and elevated IL‐6 accumulation (Figure 8A). Quantitative analyses showed higher myocardial injury indices after RASGRP1 overexpression, and several of these changes were partially improved by S1P administration (Figure 8B–D).

**Figure 9:**
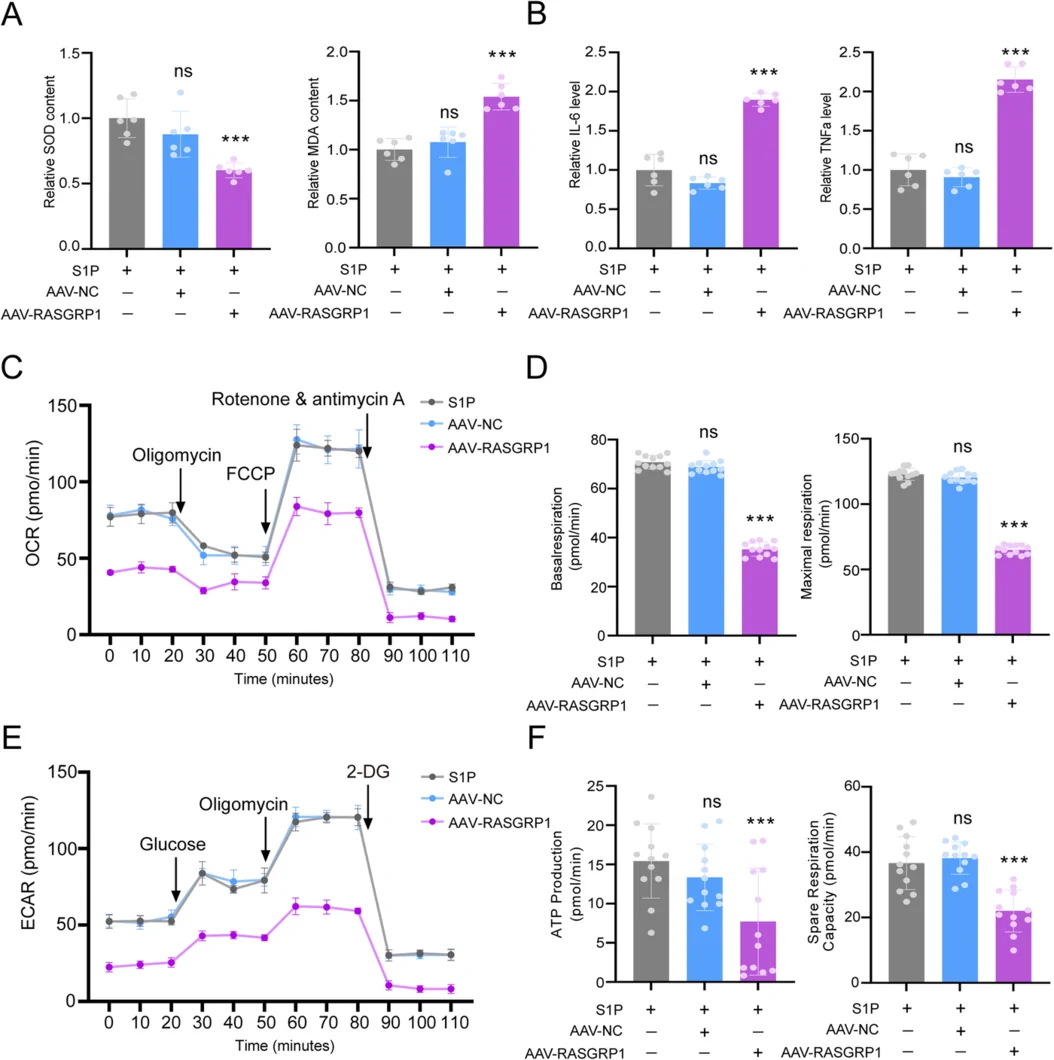
S1P rescues RASGRP1‐mediated oxidative stress and mitochondrial dysfunction. (A, B) Myocardial antioxidant enzyme activities (SOD, CAT, GSH‐Px) and MDA levels. (C) Mitochondrial OCR trace. (D) Quantification of basal and maximal respiration. (E) ECAR trace. (F) Quantification of glycolytic parameters. Data are presented as mean ± SD, with **p < 0.01, ***p < 0.001 versus AAV‐ NC + SIP group.

RASGRP1 overexpression exacerbated LPS‐induced cardiac dysfunction, as indicated by further reductions in EF and FS. S1P treatment significantly restored cardiac function in RASGRP1‐overexpressing animals and reduced serum myocardial injury markers (CK‐MB, LDH, cTnT) and pro‐inflammatory cytokines (IL‐6, TNF‐α) while increasing anti‐inflammatory IL‐10. Additionally, S1P alleviated RASGRP1‐induced redox imbalance, restoring SOD and CAT activities and reducing MDA levels (Figures 8E–I, 9A,B, and S6). Furthermore, metabolic profiling using Seahorse analysis showed that RASGRP1 overexpression markedly impaired mitochondrial oxidative phosphorylation, as reflected by reduced basal and maximal respiration and ATP production, and also suppressed glycolytic capacity. These metabolic deficits were effectively rescued by S1P treatment (Figures 9C–F and S7A,B). Therefore, RASGRP1 exacerbates myocardial injury, whereas S1P efficiently counteracts its deleterious effects.

### 3.7 Functional Effects of S100A9‐R101Q Support a Contribution of Arg101 to S1P‐Associated Responses

**Figure 10:**
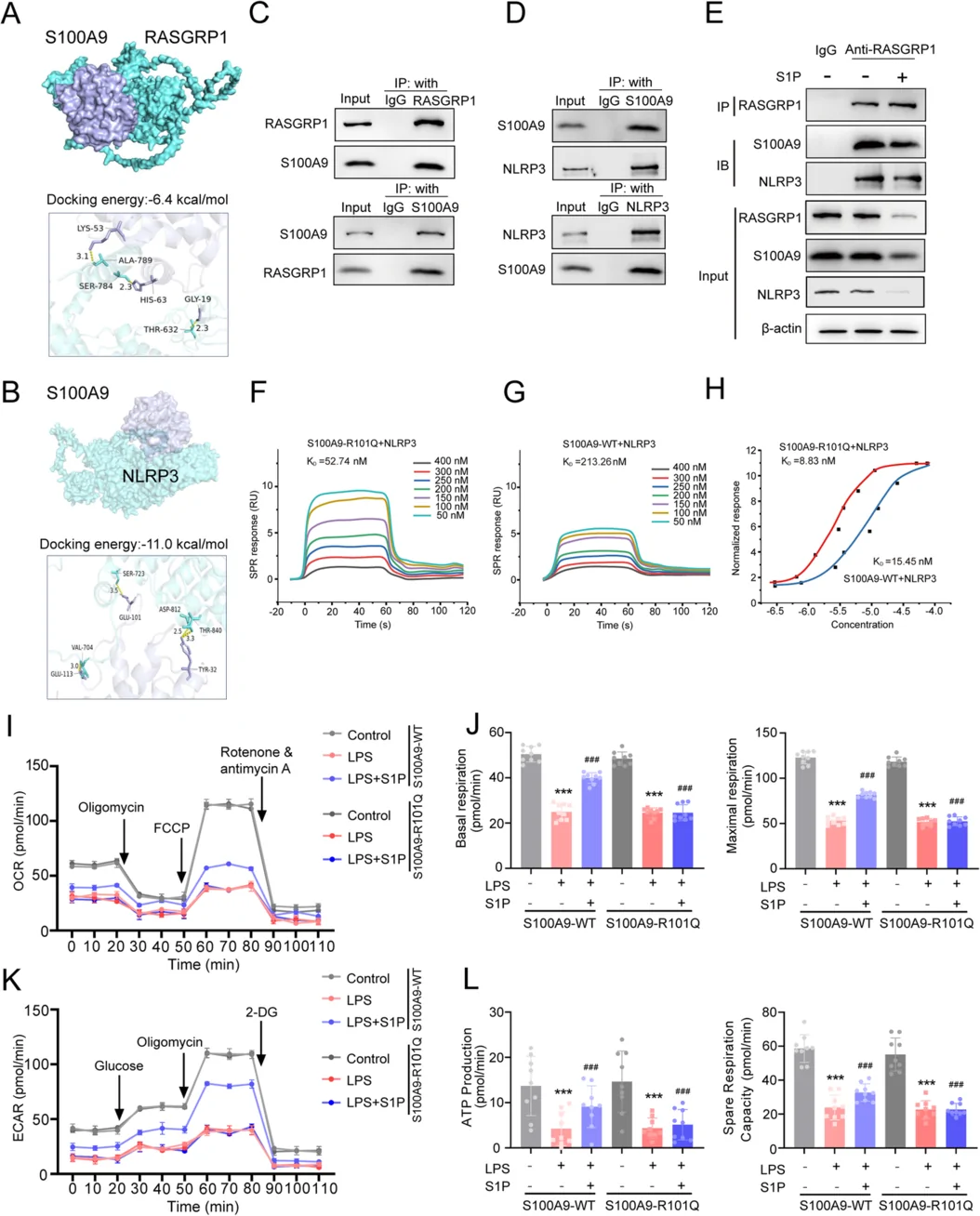
Predicted S100A9 Arg101 interface and functional effects of the S100A9‐R101Q mutation. (A) Molecular docking model of the S100A9–RASGRP1 complex. The predicted docking energy was −6.4 kcal/mol, and representative residues involved in the predicted protein–protein interface are shown. (B) Molecular docking model of the S100A9–NLRP3 complex, with a predicted docking energy of −11.0 kcal/mol. Representative interacting residues and predicted interaction distances are indicated. (C) Co‐IP analysis of the association between RASGRP1 and S100A9. RASGRP1 or S100A9 was immunoprecipitated with the indicated antibodies, followed by immunoblotting for the associated protein. IgG served as the negative control. (D) Reciprocal Co‐IP analysis of the association between S100A9 and NLRP3. (E) Co‐IP analysis of RASGRP1‐associated S100A9 and NLRP3 in the absence or presence of S1P. RASGRP1 was immunoprecipitated using an anti‐RASGRP1 antibody, and the precipitated complexes were analyzed by immunoblotting for RASGRP1, S100A9, and NLRP3. Corresponding input proteins and β‐actin are shown. (F, G) Representative SPR sensorgrams showing concentration‐dependent binding of NLRP3 to S100A9‐R101Q (F) and S100A9‐WT (G) over the indicated concentration range. Apparent kinetic K_D values are indicated. (H) Concentration–response analysis of S100A9‐R101Q and S100A9‐WT binding to NLRP3, with the fitted apparent K_D values indicated. (I) Seahorse extracellular flux analysis of mitochondrial OCR in S100A9‐WT‐ and S100A9‐R101Q‐expressing cells subjected to the indicated treatments. (J) Quantification of basal and maximal mitochondrial respiration. (K) ECAR profiles determined by Seahorse glycolytic stress analysis following sequential addition of glucose, oligomycin, and 2‐deoxy‐d‐glucose (2‐DG). (L) Quantification of ATP production and spare respiratory capacity. Data are presented as mean ± SD, with **p < 0.01, ***p < 0.001.

To clarify the role of S100A9 within the RASGRP1–NLRP3‐associated signaling network, we first performed molecular docking analyses. S100A9 showed favorable predicted interactions with both RASGRP1 (docking energy: −6.4 kcal/mol) and NLRP3 (−11.0 kcal/mol) (Figure 10A,B). Co‐immunoprecipitation assays further supported associations between RASGRP1 and S100A9, as well as between S100A9 and NLRP3, and showed that these proteins could be recovered within RASGRP1‐associated complexes (Figure 10C–E). To further investigate the functional contribution of Arg101, we generated an S100A9‐R101Q mutant. SPR analysis demonstrated measurable binding of both S100A9‐WT and S100A9‐R101Q to NLRP3, indicating that the R101Q substitution altered the interaction characteristics rather than completely abolishing S100A9–NLRP3 binding (Figure 10F–H).

Functional assays showed that LPS treatment induced marked oxidative stress, as evidenced by increased MDA levels and decreased SOD, CAT, and GSH‐Px activities, and promoted an inflammatory response characterized by increased IL‐6, TNF‐α, and IL‐1β levels together with reduced IL‐10 in S100A9‐WT cells (Figure S8A,B). S1P partially reversed these LPS‐induced alterations in S100A9‐WT cells, whereas its protective effects were markedly diminished in S100A9‐R101Q cells. These findings suggest that Arg101 contributes to the S100A9‐dependent cellular response to S1P. Consistent with these observations, mitochondrial functional analysis showed that LPS impaired both oxidative phosphorylation and glycolytic function, as reflected by reductions in basal and maximal respiration, ATP production, spare respiratory capacity, and extracellular acidification rate (Figures 10I–L and S8C). S1P substantially improved these metabolic parameters in S100A9‐WT cells, whereas this recovery was markedly attenuated in cells expressing S100A9‐R101Q. Collectively, these results indicate that Arg101 contributes to S100A9‐associated signaling and is important for the full protective effects of S1P against LPS‐induced oxidative stress, inflammation, and mitochondrial metabolic dysfunction.

### 3.8 S100A9‐R101Q Weakens S1P‐Associated Cardioprotective Responses In Vivo

**Figure 11:**
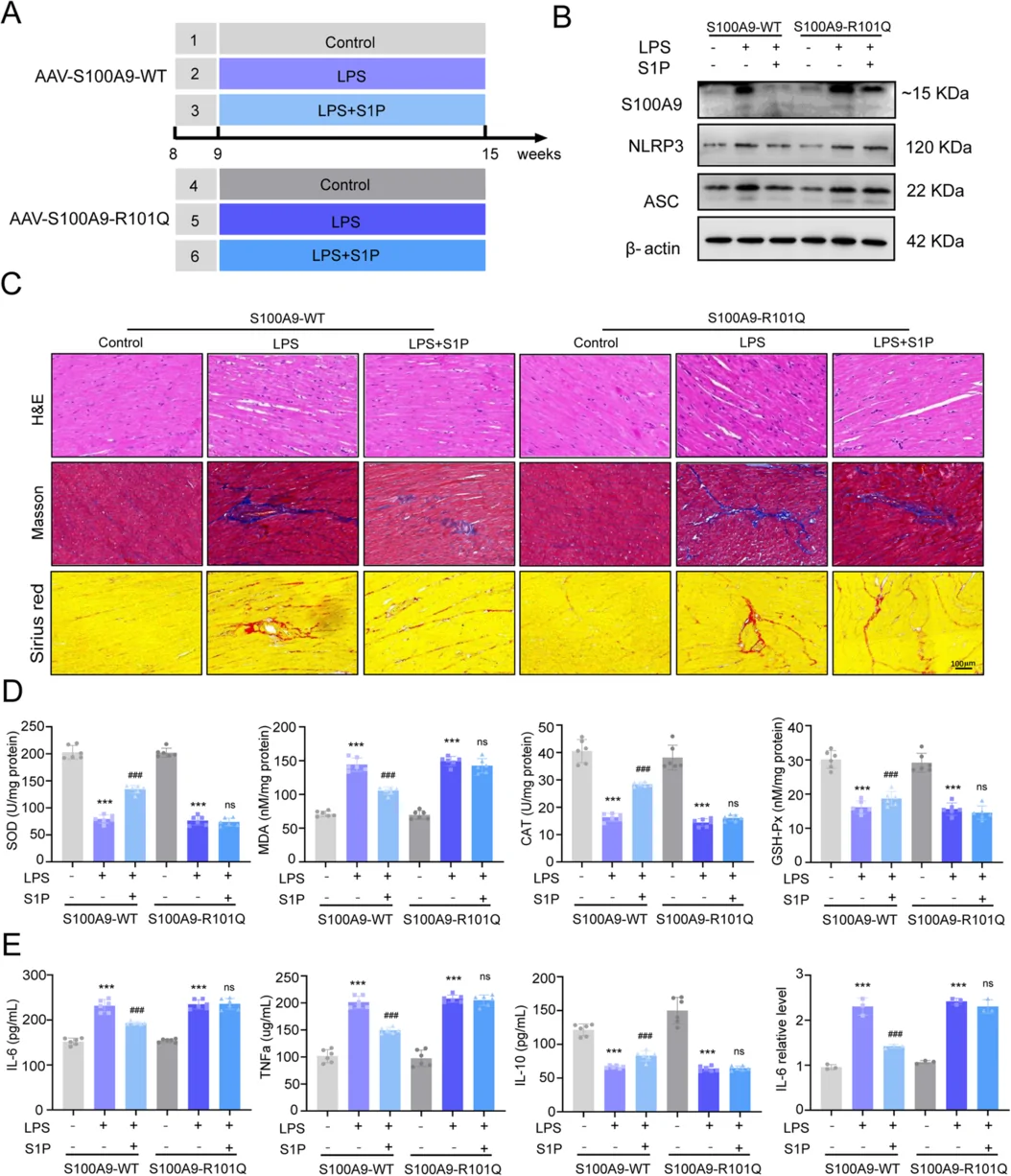
S100A9‐R101Q weakens several S1P‐associated cardioprotective responses in vivo. (A) Experimental timeline of AAV‐mediated overexpression of S100A9‐WT or R101Q in LPS‐induced inflammatory cardiac injury with S1P treatment. (B) Western blot analysis of S100A9, NLRP3, ASC, and β‐actin in myocardial tissue. (C) Representative myocardial sections stained with H&E, Masson's trichrome, and Sirius Red. (D) Myocardial antioxidant enzyme activities (SOD, CAT, GSH‐Px) and MDA levels. (E) Serum cytokine levels (IL‐6, TNF‐α, IL‐10). Data were quantified and presented as mean ± SD. Statistical significance: **p < 0.01, ***p < 0.001.

To validate the in vivo role of the S100A9 Arg101 residue in S1P‐mediated cardioprotection, we overexpressed either wild‐type S100A9 (S100A9‐WT) or the R101Q mutant via AAV in an LPS‐induced inflammatory cardiac injury model. Western blot analysis confirmed S100A9 overexpression. S1P reduced LPS‐associated increases in NLRP3 and ASC in the S100A9‐WT context, whereas this effect was attenuated in S100A9‐R101Q animals (Figure 11A,B).

Histopathological examination revealed that S1P attenuated LPS‐ associated myocardial inflammatory injury, collagen deposition, and remodeling in S100A9‐WT animals, while several of these improvements were diminished in S100A9‐R101Q animals (Figure 11C). Consistently, S1P restored antioxidant enzyme activities (SOD, CAT, GSH‐Px) and reduced MDA in S100A9‐WT rats, whereas these benefits were significantly blunted in the mutant group (Figure 11D). Similarly, S1P effectively suppressed pro‐inflammatory cytokines (IL‐6, TNF‐α) and elevated IL‐10 in S100A9‐WT rats, but these effects were markedly impaired in S100A9‐R101Q rats (Figure 11E). Therefore, Arg101 of S100A9 may contribute to S1P‐mediated cardioprotection in vivo, linking this residue to the regulation of inflammation, oxidative stress, and cardiac injury.

## 4 Discussion

In the present study, we integrated myocarditis‐associated transcriptomic analyses with an LPS‐induced inflammatory cardiac injury model to examine the relationship between S1P and macrophage‐associated RASGRP1–S100A9–NLRP3 signaling. The bioinformatic analyses prioritized RASGRP1 as a macrophage‐enriched inflammation‐associated candidate, whereas the LPS model provided a controlled experimental system for intense innate immune activation, oxidative stress, myocardial injury, and subsequent remodeling. S1P improved cardiac structure and function in vivo and attenuated inflammatory, oxidative, and metabolic abnormalities in RAW264.7 macrophages. Orthogonal SPR, MST, CETSA, and DARTS assays supported S1P–RASGRP1 target engagement, while Co‐IP and functional perturbation experiments supported associations among components of the broader inflammatory network. These findings are therefore interpreted as evidence of an S1P‐associated protective phenotype linked to RASGRP1‐centered macrophage signaling, not as proof of a fully established linear cascade.

The transcriptomic analysis also required careful clarification. The value 17,962 represents the total number of gene‐level variables subjected to differential‐expression testing in GSE35182 rather than the number of significant DEGs. After the predefined statistical thresholds were applied, 42 genes were identified as significantly differentially expressed. RFE, RF, and LASSO were used as machine learning‐assisted feature‐selection tools to prioritize candidate genes rather than as autonomous target‐identification methods. Importantly, GSE35182 is a mouse CVB3 myocarditis dataset. Thus, the bioinformatic component provides disease‐associated candidate prioritization, whereas the LPS experiments test the behavior of the selected inflammatory network in a distinct endotoxemia‐associated injury context.

Macrophages are increasingly recognized as active participants in myocardial inflammation (Francisco and Del Re 2023). In the present study, scRNA‐seq analysis showed predominant RASGRP1 enrichment in macrophages, and RAW264.7 macrophages were the principal cellular model used for mechanistic experiments. Accordingly, the RASGRP1–S100A9–NLRP3‐related findings should be interpreted as macrophage‐associated inflammatory signaling that may contribute to a tissue‐level cardioprotective phenotype. The present data do not establish an in vivo macrophage‐specific RASGRP1 mechanism. Although cardiomyocytes are direct targets of inflammatory injury in vivo, additional macrophage‐marker co‐localization, lineage‐specific manipulation, or macrophage‐specific genetic models would be required to establish the cellular origin and in vivo contribution of RASGRP1 more definitively. S100A8/A9, a myeloid‐derived alarmin complex, can amplify inflammatory signaling through pattern‐recognition receptors including TLR4 and RAGE and has been shown to promote TLR4‐dependent priming of the NLRP3 inflammasome (Sreejit et al. 2020; Vogl et al. 2007). Our Co‐IP data support associations among RASGRP1, S100A9, and NLRP3‐related proteins, and overexpression of RASGRP1 or NLRP3 attenuated several S1P‐associated protective effects. However, these observations do not establish the exact upstream‐downstream order of the three molecules. Systematic loss‐of‐function, reciprocal rescue, and epistasis experiments are still required to determine whether RASGRP1 regulates S100A9, whether S100A9 is necessary for RASGRP1‐associated NLRP3 activation, and whether parallel inflammatory pathways contribute to the phenotype.

Our results also reveal distinctions between cardiomyocyte‐centric RASGRP1–S100A9–NLRP3 activation and inflammasome activation in other cell types. Macrophages primarily activate NLRP3 in response to intracellular stress signals such as mitochondrial damage, oxidative stress, and calcium dysregulation, whereas immune cells often rely on extracellular pathogen‐ or cytokine‐derived priming signals. This distinction may explain why broad inflammasome inhibition has shown variable efficacy across cardiac disease models and underscores the importance of cell‐type‐specific targeting strategies that directly modulate cardiomyocyte inflammatory signaling. The S100A9/NLRP3 axis has been implicated in a wide spectrum of inflammatory disorders, including sepsis, atherosclerosis, acute lung injury, and neuroinflammation (Chen et al. 2023c; Fan et al. 2024; Gong et al. 2024; Pashaei et al. 2025). In these contexts, S100A9 acts as both an extracellular inflammatory ligand and an intracellular signaling modulator, promoting NLRP3 inflammasome activation and cytokine maturation. Our study extends these observations to inflammatory cardiac injury and provides direct evidence that cardiomyocyte‐intrinsic activation of this pathway is not merely a bystander phenomenon but a driving force of myocardial inflammation.

Beyond inflammasome activation, RASGRP1 and S100A9 may modulate additional inflammatory pathways, including MAPK signaling, JAK/STAT activation, and NADPH oxidase‐mediated oxidative stress (Chen et al. 2023b; Du et al. 2022; Zha et al. 2023). Although our study focused on NLRP3 activation, it is plausible that macrophage‐derived RASGRP1–S100A9 simultaneously influences parallel inflammatory circuits, collectively exacerbating myocardial injury. This multifaceted role highlights the potential of targeting the RASGRP1–S100A9–NLRP3 axis as a nodal therapeutic strategy. Inflammasome activation is closely linked to pyroptosis, a lytic form of programmed cell death mediated by gasdermin D (GSDMD) (Burdette et al. 2021). Previous studies have demonstrated that NLRP3–caspase‐1 signaling leads to GSDMD cleavage and pore formation, resulting in inflammatory cell death (Xu and Núñez 2023). Our findings suggest that RASGRP1–S100A9‐driven NLRP3 activation may contribute to cardiomyocyte pyroptosis in inflammatory cardiac injury, amplifying local inflammation through the release of intracellular contents. However, S100A9 can also promote inflammation independently of pyroptosis, emphasizing the complexity of its biological functions in macrophages.

A major contribution of this study is the identification of S1P as a regulator of the RASGRP1–S100A9–NLRP3 axis in inflammatory cardiac injury. S1P suppresses RASGRP1 and S100A9 expression in macrophages, attenuating downstream NLRP3 inflammasome activation and thereby limiting intrinsic inflammatory amplification. Although S1P is widely recognized for its roles in immune cell trafficking, vascular integrity, and cardioprotection (Vestri et al. 2017), its involvement in cardiomyocyte inflammasome regulation has remained incompletely understood. Our results suggest that, in inflammatory cardiac injury, S1P predominantly engages protective and anti‐inflammatory receptor pathways, providing mechanistic insight into how lipid mediators fine‐tune innate immune signaling within non‐immune cells, with implications for other inflammatory cardiomyopathies (Obinata and Hla 2019).

Several limitations of this study should be acknowledged. First, the in vivo study used a single LPS challenge, which predominantly models endotoxemia‐associated innate immune activation and systemic inflammatory cardiac injury. Although this model reproduces inflammatory mediator release, oxidative stress, myocardial structural injury, cardiac‐injury‐marker elevation, and impaired cardiac function, it does not reproduce viral infection and replication, cardiac antigen‐specific adaptive immunity, or autoimmune‐mediated myocardial injury. The 6‐week treatment and follow‐up period allowed assessment of post‐inflammatory collagen deposition and remodeling after the initial insult, but it does not imply 6 weeks of continuous endotoxemia. Validation in experimental autoimmune myocarditis, appropriate viral myocarditis models, and ultimately human myocardial samples will be required before the findings can be generalized to classical myocarditis.

Second, the precise S1P receptor subtype(s) mediating these effects were not fully dissected, and future studies with receptor‐specific agonists, antagonists, or cardiomyocyte‐specific knockouts are needed. S1P is an endogenous bioactive lipid whose biological effects are primarily mediated through S1PR1–S1PR5. Although the SPR, MST, CETSA, and DARTS results support an interaction between S1P and RASGRP1, they do not exclude the involvement of canonical S1P receptor‐dependent signaling. Further studies using receptor‐selective agonists, antagonists, or genetic approaches are required to determine the relative contributions of RASGRP1 engagement and individual S1P receptor subtypes to the observed cardioprotective effects.

Third, our in vivo experiments relied primarily on a single inflammatory cardiac injury model. While this model recapitulates key features of inflammatory cardiomyopathy, validation in additional experimental systems, including viral inflammatory cardiac injury models, would strengthen the generalizability of our findings. A limitation of this study is that the hierarchical relationship among RASGRP1, S100A9, and NLRP3 remains incompletely defined. Although functional experiments support their involvement in the S1P‐associated protective phenotype, systematic loss‐of‐function, rescue, and epistasis studies were not performed. Future studies using targeted genetic manipulation and epistasis analyses are needed to clarify their upstream–downstream relationships.

Fourth, although macrophages were the primary focus of this study, other immune and non‐immune cardiac cell types may also contribute to S1P‐mediated immunoregulation. Single‐cell and spatial transcriptomic approaches could further clarify intercellular communication within the inflamed myocardium. Although our scRNA‐seq analysis showed that RASGRP1 was predominantly enriched in macrophages, its cell‐type‐specific localization in vivo was not further confirmed by co‐immunofluorescence staining or lineage‐specific approaches. Future studies using macrophage‐specific genetic models are therefore needed to establish the precise contribution of macrophage‐derived RASGRP1 to inflammatory cardiac injury.

Finally, while our data support the therapeutic potential of targeting the RASGRP1–S100A9–NLRP3 axis via S1P, translational studies are required to assess clinical feasibility and safety. The relatively small animal sample size (*n* = 6 per group) represents an additional limitation of this study and may restrict the statistical power and generalizability of the findings. Future studies with larger, independently replicated cohorts are warranted to confirm the robustness of these results.

## 5 Conclusions

In summary, S1P attenuates LPS‐induced inflammatory cardiac injury in association with modulation of macrophage‐associated RASGRP1–S100A9–NLRP3 signaling. SPR, MST, CETSA, and DARTS support direct S1P–RASGRP1 target engagement, while Co‐IP and functional perturbation experiments support involvement of the broader inflammatory network. However, the complete signaling hierarchy, the precise residue‐level binding mechanism, and the relative contribution of canonical S1PR signaling remain unresolved. Because the LPS model primarily reflects endotoxemia‐associated inflammatory cardiac injury, validation in autoimmune and viral myocarditis models and in human myocardial samples is required before extending these conclusions to classical myocarditis.

## Author Contributions

Conceptualization, methodology, data curation, writing—original draft: Chaofu Yue, Qiaolin Li, Chunyan Li, and Taoxian Yang. Methodology, data curation, software, validation, investigation: Xian Huang, Feng Yue, Qiuyu Long, Rong Li, Rong Lei, Qingsong Ma, Caimei Hu, Qian Yang, Yongjun Yan, Yuan Liu, and QinYong Yan. Supervision, conceptualization, validation, writing—original draft, writing—review and editing: Chaofu Yue and Mei Yang. All authors read and approved the final manuscript. All authors contributed to the study conception and design. Chaofu Yue, Qiaolin Li, Chunyan Li, and Taoxian Yang contributed equally to this work and are co‐first authors. Chaofu Yue and Mei Yang are corresponding authors.

## Ethics Statement

All animal procedures were conducted in accordance with the National Institutes of Health Guide for the Care and Use of Laboratory Animals (NIH publication, 8th Edition, 2011, USA). Moreover, the procedures were approved by the Kunming Medical University (kmmu20230974).

## Consent

All authors give their full consent to publish the present article.

## Conflicts of Interest

The authors declare no conflicts of interest.

## Supporting information


Supporting File 1

Supporting File 2

## Data Availability

The data used in this study are available and will be provided by the corresponding author upon reasonable request.
