# Supplementary material for: Sphingosine‐1‐Phosphate Attenuates LPS‐Induced Inflammatory Cardiac Injury in Association With RASGRP1–S100A9–NLRP3 Signaling
Source: Drug Dev Res. 2026 Sep 22;87(7):e70391. doi: 10.1002/ddr.70391 (PMC13595488; doi:10.1002/ddr.70391)
Supplement: Supplementary file 1 [file DDR-87-e70391-s002.docx]

**Supplementary Materials**

**Sphingosine-1-Phosphate Attenuates LPS-Induced Inflammatory Cardiac Injury in Association with RASGRP1–S100A9–NLRP3 Signaling**

Chaofu Yue^1,#*^, Qiaolin Li^1, #^, Chunyan Li^2, #^, Taoxian Yang^3, #^, Xian Huang^1^, Feng Yue^1^, Qiuyu Long^1^, Rong Li^1^, Rong Lei^1^, Qingsong Ma^1^, Caimei Hu^1^, Qian Yang^1^, Yongjun Yan^1^, Yuan Liu^1^, QinYong Yan^1^, Mei Yang^1, *^

1. Department of Intensive Care Unit, Affiliated Qujing Hospital of Kunming Medical University, Qujing Central Hospital of Yunnan Province, Qujing, 655000, Yun Nan, China.

2. Department of Endocrinology and Metabolism, Affiliated Qujing Hospital of Kunming Medical University, Qujing Central Hospital of Yunnan Province, Qujing, 655000, Yun Nan, China.

3. Nursing department, Affiliated Qujing Hospital of Kunming Medical University, Qujing Central Hospital of Yunnan Province, Qujing, 655000, Yun Nan, China.

^#^ These authors contributed equally.

*Correspondence authors: Dr. Chaofu Yue, [yueaaa0@163.com](mailto:yueaaa0163@.com), Dr. Mei Yang, [ym780926@qq.com](mailto:ym780926@qq.com) , Department of Intensive Care Unit, Affiliated Qujing Hospital of Kunming Medical University, Qujing Central Hospital of Yunnan Province, Qujing, 655000, Yun Nan, China. Telephone Number: 0874-6661505

**Figure S1**

**Figure S1: Molecular docking models of additional top candidate compounds with RASGRP1.** The top 9 small-molecule compounds obtained from virtual screening were individually docked to RASGRP1., and their predicted binding modes and docking scores were analyzed.

**Figure S2**

**Figure S2. S1P modulates cytokine profiles in LPS-induced associated inflammatory cardiac injury.** (A–C) Serum levels of (A) IL-6, (B) TNF-α, and (C) IL-10 across experimental groups. Data presented as mean ± SD, ****p* < 0.001 versus Control group, , ##*p* < 0.01, ###*p* < 0.001 versus LPS group.

**Figure S3**

**Figure S3. S1P reduces myocardial injury markers and improves mitochondrial metabolism.** (A) CK‑MB, LDH, and cTnT levels in supernatant. (B) Mitochondrial respiratory parameters: basal respiration, maximal respiration, ATP production, spare capacity. (C) Glycolytic parameters: glycolysis, glycolytic capacity, glycolytic reserve. Data presented as mean ± SD, ****p* < 0.001 versus Control group, ###*p* < 0.001 versus LPS group.

**Figure S4**

**Figure S4. S1P dose-dependently inhibits the RASGRP1-NLRP3 inflammasome pathway.** Western blot analysis of RASGRP1, S100A9, NLRP3, and ASC expression. Data presented as mean ± SD, ****p* < 0.001 versus Control group, ###*p* < 0.001 versus LPS group.

**Figure S5**

**Figure S5. AAV-mediated NLRP3 overexpression exacerbates LPS-induced impairment of cardiac glycolytic function.** Measurements of glycolysis and glycolytic capacity from control and AAV‑NLRP3‑overexpressing groups following LPS challenge, highlighting the suppressive effect of NLRP3 overexpression on cardiac glycolytic metabolism. Data presented as mean ± SD, ***p* < 0.01, and ****p* < 0.001 versus AAV-NC+S1P group.

**Figure S6**

**Figure S6. RASGRP1 overexpression abrogates S1P-mediated attenuation of oxidative stress in LPS-induced associated inflammatory cardiac injury**. Data presented as mean ± SD, ****p* < 0.001 versus S1P group, ###*p* < 0.001 versus AAV-NC+S1P group.

**Figure S7**

**Figure S7. RASGRP1 overexpression abrogates S1P-mediated improvements in oxidative stress, cytokine balance, and glycolytic function in LPS-induced associated inflammatory cardiac injury.** (A) Dot plots showing relative levels of oxidative stress markers (CAT content, GSH-Px content) and cytokines (IL-1β, IL-10) in myocardial tissues. (B) Dot plots showing glycolytic function parameters (glycolysis, glycolytic capacity, glycolytic reserve) measured via Seahorse assay in myocardial cells. Data presented as mean ± SD, ###*p* < 0.001 versus AAV-NC+S1P group.

**Figure S8**

**Figure S8. Effects of S100A9-R101Q on S1P-mediated regulation of oxidative stress, inflammatory responses, and glycolytic function.** (A) Quantification of oxidative stress-related indices in S100A9-WT- and S100A9-R101Q-expressing cells subjected to the indicated treatments, including relative SOD content, MDA content, CAT activity, and GSH-Px content. (B) Relative levels of the inflammatory cytokines IL-6, TNF-α, and IL-1β and the anti-inflammatory cytokine IL-10 in S100A9-WT- and S100A9-R101Q-expressing cells following LPS and/or S1P treatment. (C) Seahorse extracellular flux-derived quantification of glycolysis, glycolytic reserve, and glycolytic capacity in S100A9-WT- and S100A9-R101Q-expressing cells under the indicated treatment conditions. Data presented as mean ± SD, ****p* < 0.001 versus control group; and ###*p* < 0.001 versus LPS-treated group.
